# CAP2 promotes gastric cancer metastasis by mediating the interaction between tumor cells and tumor-associated macrophages

**DOI:** 10.1172/JCI166224

**Published:** 2023-11-01

**Authors:** Guohao Zhang, Zhaoxin Gao, Xiangyu Guo, Ranran Ma, Xiaojie Wang, Pan Zhou, Chunlan Li, Zhiyuan Tang, Ruinan Zhao, Peng Gao

**Affiliations:** Department of Pathology, Qilu Hospital, and Department of Pathology, School of Basic Medical Sciences, Shandong University, Jinan, Shandong, China.

**Keywords:** Gastroenterology, Oncology, Cytokines, Macrophages, Oncogenes

## Abstract

The metastasis of cancer cells is the main cause of death in patients with gastric cancer (GC). Mounting evidence has demonstrated the vital importance of tumor-associated macrophages in promoting tumor invasion and metastasis; however, the interaction between tumor cells and macrophages in GC is largely unknown. In this study, we demonstrated that cyclase-associated protein 2 (CAP2) was upregulated in GC, especially in cases with lymph node metastasis, and was correlated with a poorer prognosis. The transcription factor JUN directly bound to the promoter region of *CAP2* and activated *CAP2* transcription. The N-terminal domain of CAP2 bound to the WD5 to WD7 domains of receptor for activated C kinase 1 (RACK1) and induced M2 macrophage polarization by activating the SRC/focal adhesion kinase (FAK)/ERK signaling pathway, which resulted in IL-4 and IL-10 secretion. Polarized M2 macrophages induced premetastatic niche formation and promoted GC metastasis by secreting TGFB1, which created a TGFB1/JUN/CAP2 positive-feedback loop to activate CAP2 expression continuously. Furthermore, we identified salvianolic acid B as an inhibitor of CAP2, which effectively inhibited GC cell invasion capabilities by suppressing the SRC/FAK/ERK signaling pathway. Our data suggest that CAP2, a key molecule mediating the interaction between GC cells and tumor-associated macrophages, may be a promising therapeutic target for suppressing tumor metastasis in GC.

## Graphical abstract

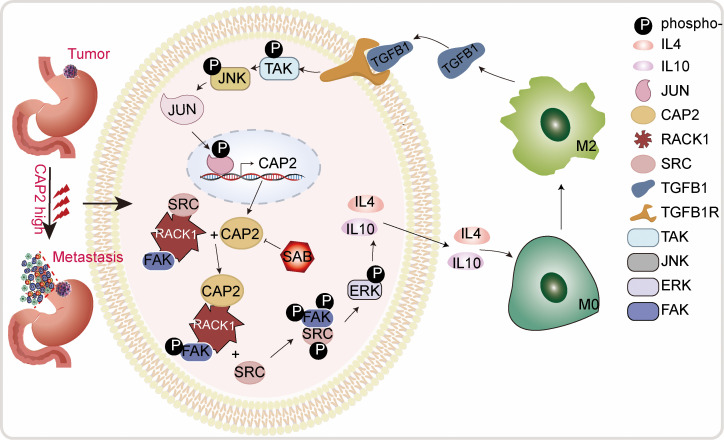

## Introduction

Gastric cancer (GC) is the fifth most common cancer worldwide (1). Although substantial advances have been achieved in the diagnosis and treatment of GC in the past few years, the mortality of patients with GC remains high, especially in cases of patients with advanced-stage disease or metastasis (2, 3). The cause of death in most cancers is not the primary tumor but tumor metastasis, which causes approximately 90% of cancer-related deaths (4). Thus, it is vital to identify the mechanisms underlying tumor metastasis.

The tumor microenvironment is a complex cellular environment consisting of multiple types of cells, including macrophages, fibroblasts, dendritic cells, and lymphocytes. Macrophages are abundant at all stages of tumor progression (5). The “classically activated” M1 macrophages are induced by proinflammatory stimuli and participate in antitumor reactions. However, cytokines such as IL-4, IL-13, IL-10, and TGFB1 support “alternatively activated” M2 macrophages (6). Tumor-associated macrophages (TAMs) are usually M2 subtypes and play protumor roles (7). Nevertheless, little is known about the mechanism underlying the crosstalk between TAMs and GC cells.

In our previous study, microarray analysis (GEO GSE72307) was performed to compare differential gene expression in primary GC samples with and without lymph node metastasis (LNM). The results showed that *CAP2* was upregulated in GC tissues with LNM. The expression of cyclase-associated protein 2 (CAP2) is altered in several human cancers (8–10). However, the biological functions and underlying mechanisms of CAP2 in GC remain poorly understood.

In this study, we evaluated the expression and function of CAP2 in GC and elucidated the mechanisms underlying the role of the CAP2-mediated interaction between tumor cells and macrophages in promoting GC metastasis. CAP2 may be a potential prognostic biomarker and therapeutic target in GC.

## Results

### CAP2 is upregulated in human GC tissues and is associated with a poor prognosis for patients with GC.

**Figure 1:**
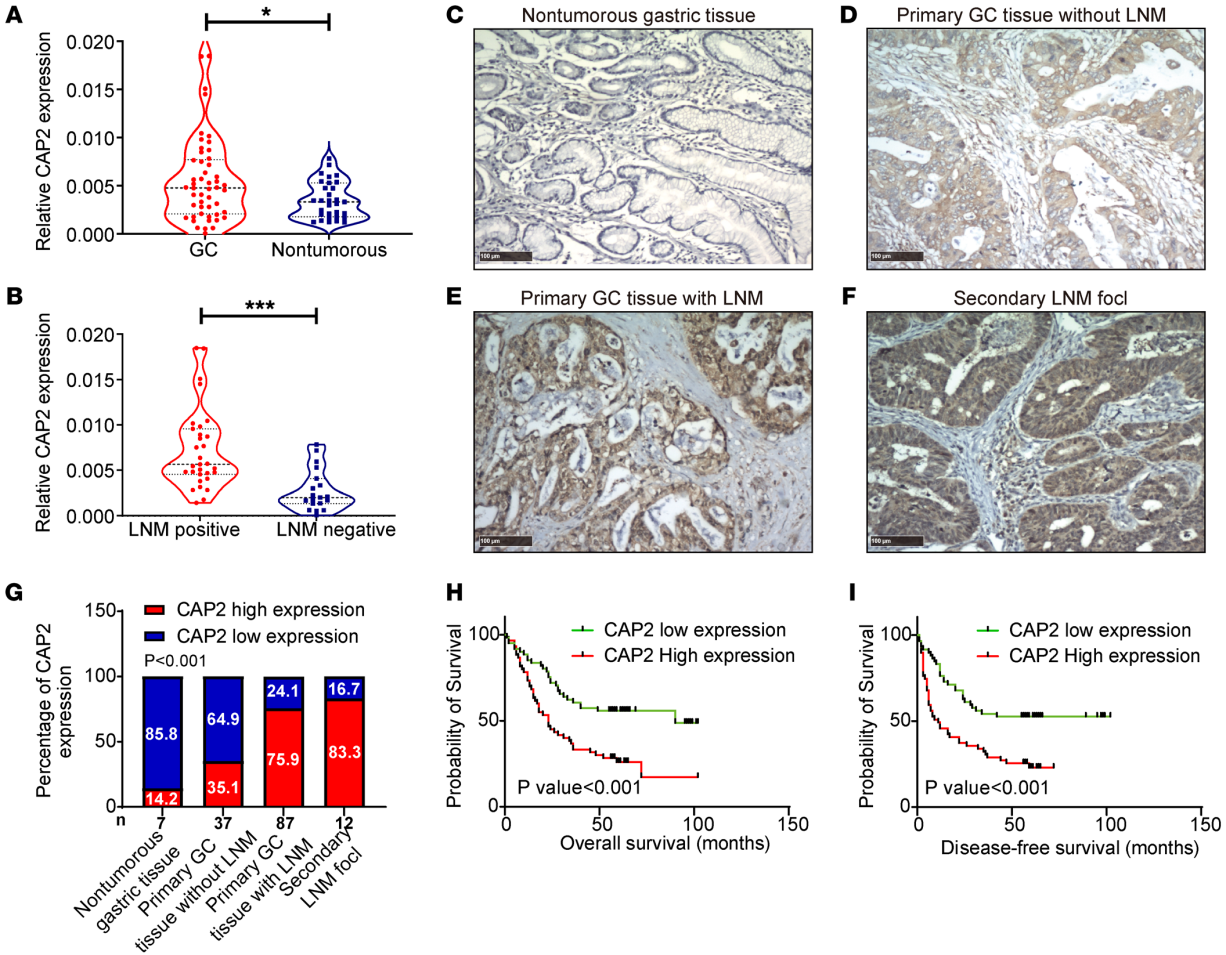
CAP2 is upregulated in human GC tissues and is associated with a poor prognosis. (**A**) *CAP2* mRNA expression in GC tissues and nontumorous gastric tissues was detected with reverse transcriptase PCR (RT-PCR) (*n*[GC] = 50, *n*[Nontumorous] = 31). (**B**) *CAP2* mRNA expression in GC tissues with and without LNM was detected by qPCR (*n*[positive] = 31, *n*[negative] = 19). The violin plot shows the mean and interquartile range, and the width shows the probability density. (**C**–**F**) Expression of CAP2 protein in GC paraffin-embedded tissue was detected by IHC. Representative images of CAP2 expression in normal gastric mucosa and GC tissues of different grades. Original magnification, ×100; scale bars: 100 μm. (**G**) Percentage of the high and low CAP2 expression levels in GC samples with different metastatic status and in normal gastric mucosal samples. (**H** and **I**) Kaplan-Meier curves of overall survival and disease-free survival for CAP2 expression. The cutoff value was obtained using the median analysis. (The numbers of patients with high and low CAP2 expression were equal. *n*[low] = 61, *n*[high] = 61.) Two-tailed unpaired Student’s *t* test (**A** and **B**), χ^2^ test (**G**), log-rank test (**H** and **I**). *P < 0.05, ***P < 0.001.

We first determined *CAP2* mRNA expression in fresh GC tissues. The results showed that the expression level of *CAP2* in GC tissues was significantly upregulated (Figure 1A). Moreover, *CAP2* mRNA expression was further increased in GC tissues with LNM (Figure 1B). Receiver operating characteristic (ROC) curves showed that *CAP2* expression could discriminate GC tissues from nontumorous gastric tissues, and also discriminate GC tissues with LNM from tissues without LNM (Supplemental Figure 1, A and B; supplemental material available online with this article; https://doi.org/10.1172/JCI166224DS1). Next, we examined CAP2 protein expression in paraffin-embedded tissues. The expression level of CAP2 protein was higher in GC tissues, especially in tissues with LNM, than in nontumorous tissues (Figure 1, C–F). As shown in Figure 1G, we performed a statistical analysis of CAP2 expression in GC samples, and the results indicated that CAP2 had the lowest proportion of high expression in the normal gastric mucosa (14.2%) and the highest proportion of high expression in metastatic lymph nodes (83.4%). Analysis of the association between CAP2 expression and clinicopathological parameters demonstrated that high CAP2 expression was positively correlated with clinical stage, distant metastasis, LNM, and tumor-node-metastasis (TNM) grade (Table 1). In addition, we selected the cutoff values according to the median (Figure 1, H and I) and ROC curves (Supplemental Figure 1, C and D) of the immunohistochemistry scores of patient samples, and the results showed that patients with higher CAP2 expression had shorter disease-free survival and overall survival.

We also analyzed the mRNA expression of *CAP2* in the Gene Expression Profiling Interactive Analysis (GEPIA) database and found that *CAP2* expression was higher in GC tissues compared with normal tissues (Supplemental Figure 1E). Moreover, analyses of data in databases (GEPIA, Kaplan-Meier Plotter, and TIMER2.0) revealed that patients with higher CAP2 expression had a poorer prognosis (Supplemental Figure 1, F–H). The above results suggest that CAP2 is upregulated in metastatic GC and serves as a potential prognostic marker for GC patients.

### JUN activates CAP2 transcription.

**Figure 2:**
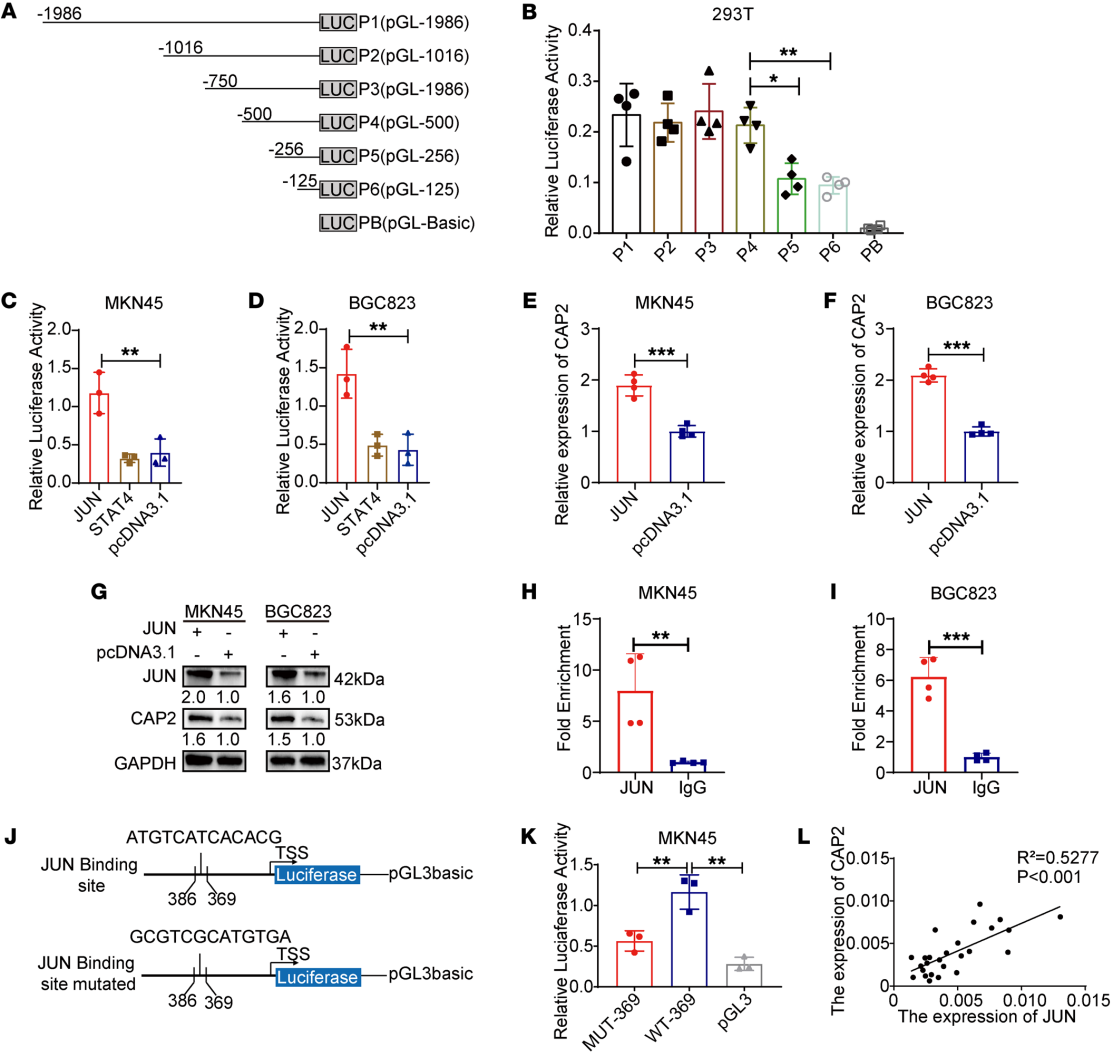
JUN activates *CAP2* transcription. (**A**) Schematic representation of truncation of the *CAP2* promoter region. (**B**) *CAP2* core promoter region was detected in 293T cells by dual-luciferase activity assay (*n* = 4). (**C** and **D**) Dual-luciferase activity assay demonstrated that JUN promoted pGL-500 promoter activity in GC cells (*n* = 3). (**E** and **F**) RT-PCR assay indicated that JUN promoted the expression of *CAP2* mRNA in GC cells (*n* = 4). (**G**) Western blot showed that JUN promoted the expression of CAP2 protein. (**H** and **I**) ChIP showed that JUN was significantly enriched in the *CAP2* promoter region in GC tissues (*n* = 4). (**J**) Schematic representation of the luciferase reporter gene of the *CAP2* promoter region and mutants (−386 to −369). (**K**) Dual-luciferase activity assays indicated that JUN binding mutants were unable to enhance pGL-500 promoter activity in MKN45 cells (*n* = 3). (**L**) mRNA expression of *JUN* and *CAP2* was determined by RT-PCR (*n* = 26). Data are presented as the mean ± SD. One-way ANOVA with Tukey’s multiple-comparison test (**B**–**D** and **K**), 2-tailed unpaired Student’s *t* test (**E**, **F**, **H**, and **I**), Pearson’s correlation (**L**). *P < 0.05, **P < 0.01, ***P < 0.001.

Given that CAP2 is highly expressed in GC, transcription factors can promote gene expression in multiple cancers. To clarify the mechanism of CAP2 expression, we conducted a promoter analysis to identify potential regulators. To identify the active promoter region of *CAP2*, we constructed pGL3-1986 and 5 truncated fragments (Figure 2A). Compared with the negative control, the pGL3-1986 group demonstrated stronger luciferase activity. The luciferase activity of pGL3-256 was significantly decreased compared with that of pGL3-500, indicating that the promoter region between –500 bp and –256 bp was the core promoter region of *CAP2* (Figure 2B and Supplemental Figure 2, A and B).

To identify transcription factors that activate *CAP2* expression, we analyzed the core promoter region of *CAP2* using the JASPAR database. The binding sites of 4 transcription factors — JUN, signal transducer and activator of transcription 4 (STAT4), E2F transcription factor 1 (E2F1), and CCAAT/enhancer-binding protein β (CEBPB) — were found. These transcription factors were individually expressed in GC cells, along with the pGL3-500 plasmid. The results showed that only JUN rather than STAT4, E2F1, or CEBPB could increase the luciferase activity of pGL3-500 (Figure 2, C and D, and Supplemental Figure 2, C and D), and JUN promoted the mRNA and protein expression of CAP2 (Figure 2, E–G). Next, chromatin immunoprecipitation (ChIP) analysis showed that JUN was enriched on the promoters of *CAP2* compared with the control group (Figure 2, H and I). The luciferase assays showed that the luciferase activity of the mutant binding sites was significantly lower than that of the wild-type (Figure 2, J and K, and Supplemental Figure 2, E and F). Then we detected the mRNA expression of *CAP2* and *JUN* in GC tissues and found that *CAP2* was positively correlated with *JUN* (Figure 2L). In addition, analyses of data in the GEPIA and TNMplot databases were consistent with our results (Supplemental Figure 2, G and H). In conclusion, JUN acts as a transcription factor to bind to the core promoter region of *CAP2* and promote its transcription.

### CAP2 promotes GC progression.

**Figure 3:**
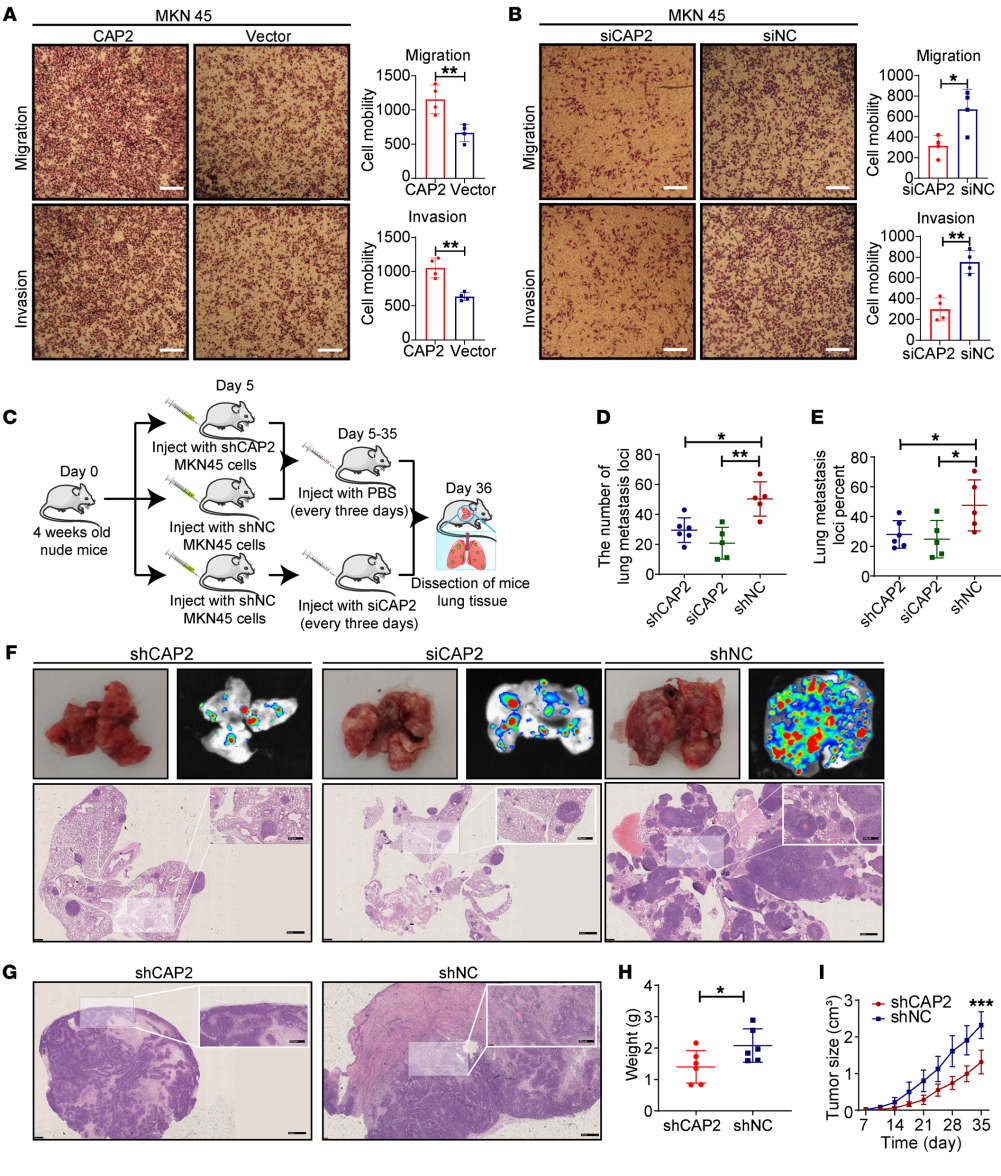
CAP2 promotes GC progression. (**A** and **B**) The migration and invasion ability of MKN45 cells was determined by Transwell assay. Original magnification, ×40; scale bar: 200 μm (*n* = 4). (**C**) GC cells were injected into the tail vein of mice to obtain lung xenografts. After LV-NC (*n* = 5) and LV-shCAP2 (*n* = 6) were injected into the tail vein of mice, the siCAP2 group (*n* = 5) was treated with siRNA every 3 days. (**D** and **E**) Number of lung transplanted tumors (**D**) and the ratio of transplanted tumor/normal lung tissue area (**E**) in nude mice. (**F**) Representative photographs of lung metastases on day 36. Scale bars: 1 mm; 500 μm (insets). (**G**) H&E staining showed that the LV-shCAP2 group had an intact capsule, while the LV-shNC group had local infiltration. Scale bars: 1 mm; 500 μm (insets). (**H** and **I**) LV-shCAP2 or negative control was used for mouse subcutaneous tumorigenesis experiments. At 36 days after the subcutaneous injection, tumor weight was measured (**H**). Tumor volumes were measured weekly, and tumor growth curves were drawn (**I**). Data are presented as the mean ± SD. Two-tailed unpaired Student’s *t* test (**A**, **B**, and **H**), 1-way ANOVA with Tukey’s multiple-comparison test (**D** and **E**), 2-way ANOVA test (**I**). *P < 0.05, **P < 0.01, ***P < 0.001.

To investigate the role of CAP2 in GC, we examined the expression levels of *CAP2* in the immortalized gastric epithelial cell line GES-1 and four GC cell lines. The results indicated that the mRNA and protein expression of CAP2 was the highest in the MKN45 cell line and the lowest in the BGC823 cell line (Supplemental Figure 3, A and B). As a result, we established overexpression and knockdown systems in 2 cell lines, MKN45 and BGC823 (Supplemental Figure 3, C–E). Transwell experiments showed that overexpression of *CAP2* promotes the migration and invasion of GC cells (Figure 3A and Supplemental Figure 3F). Conversely, knockdown of the expression of *CAP2* could inhibit the migration and invasion capabilities of GC cells (Figure 3B and Supplemental Figure 3G). Cell scratch experiments have also confirmed that knocking out CAP2 inhibits cell migration (Supplemental Figure 3, H and I). However, CCK-8 and EdU experiments showed that CAP2 did not affect GC proliferation (Supplemental Figure 4, A–H). These in vitro experiments demonstrate that CAP2 promotes migration and invasion of GC cells but has no effect on cell proliferation.

To determine whether CAP2 regulates GC in vivo, we injected GC cells transfected with lentiviral vector–shRNA–CAP2 (LV-shRNA-CAP2) or LV-shRNA-negative control (LV-shRNA-NC) into the tail vein of nude mice to observe tumor metastasis (Figure 3C). The metastases were inhibited in the LV-shCAP2 group (Figure 3, D–F). Notably, the knockdown of CAP2 with 2′-*O*-methylation–modified RNA interference significantly reduced tumor lung metastasis (Figure 3, D–F). In xenograft tumor models, tumor capsules were intact in the LV-shCAP2 group. However, tumors in the LV-NC group showed local infiltration (Figure 3G). Interestingly, unlike in in vitro experiments, the volume and weight of xenograft tumors were reduced in the LV-shCAP2 group compared with the LV-shNC group (Figure 3, H and I, and Supplemental Figure 4I). These results suggest that knocking down *CAP2* suppresses tumor growth and metastasis in vivo.

### CAP2 binds to RACK1 and activates the FAK/MEK/ERK axis.

**Figure 4:**
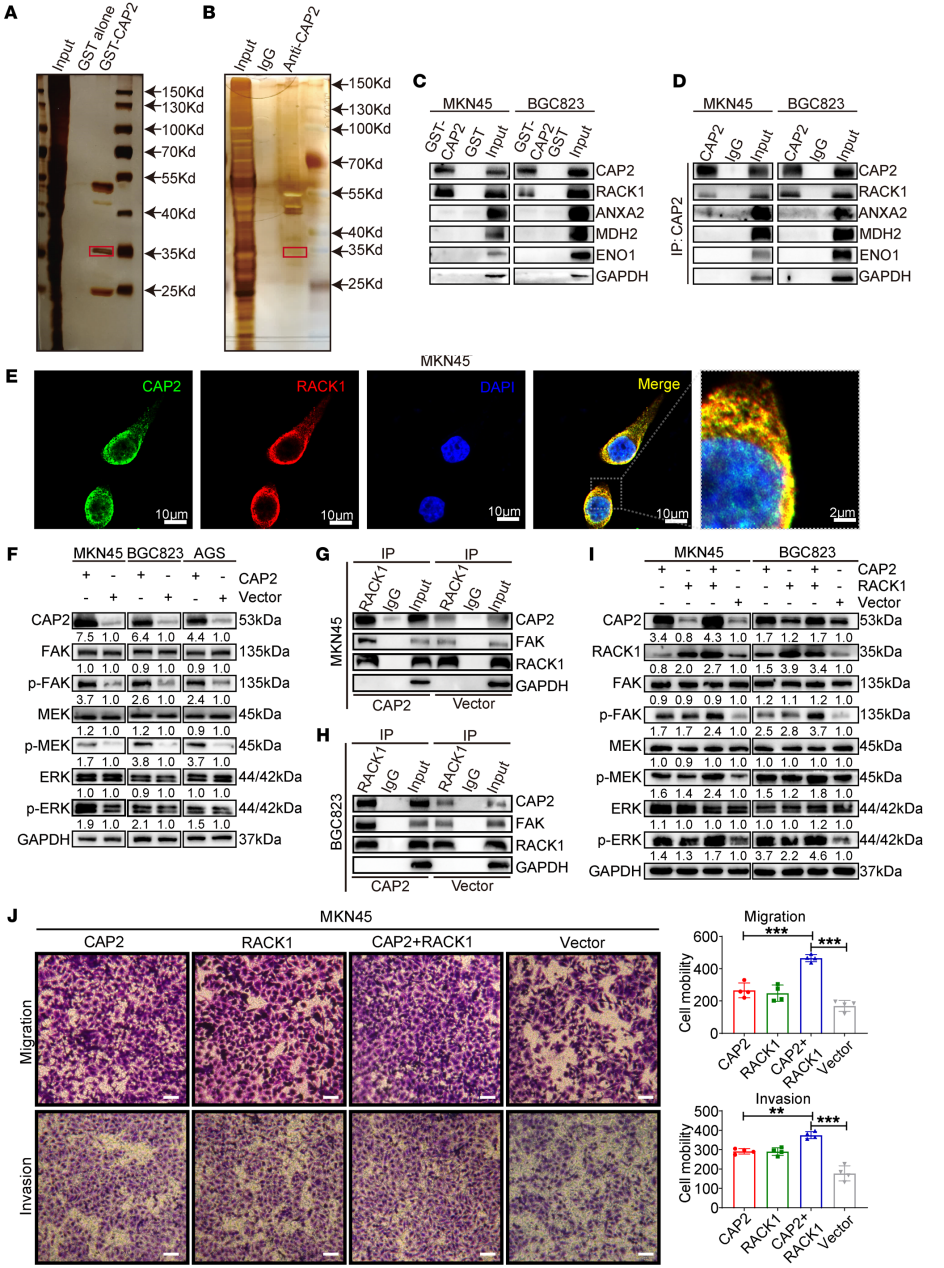
CAP2 binds to RACK1 and activates the FAK/MEK/ERK axis. (**A**) Pull-down experiments were performed on lysates from MKN45 cells using GST-CAP2 or GST-tagged proteins, followed by gel electrophoresis. Liquid chromatography–tandem mass spectrometry (LC-MS/MS) was performed on 30- to 70-kDa proteins. (**B**) Cell lysates were immunoprecipitated with anti-CAP2 or IgG. The co-IP elutions were silver-stained, and all coimmunoprecipitated proteins were analyzed by LC-MS/MS. (**C**) In vitro binding between RACK1 and GST-CAP2 was analyzed by GST pull-down assays. (**D**) Co-IP showed that RACK1 was immunoprecipitated by CAP2, rather than ANXA2, MDH2, or GAPDH. (**E**) Immunofluorescence analysis showed the CAP2/RACK1 colocalization in MKN45 cells. Scale bars: 10 μm; 2 μm (right). (**F**) Effects of CAP2 on FAK/MEK/ERK signaling pathway in GC cells were detected by Western blot. (**G** and **H**) Effects of CAP2 on the binding strength of RACK1/FAK complex were detected by co-IP assays. (**I**) Effects of CAP2 and RACK1 on FAK/MEK/ERK signaling pathway were detected by Western blot. (**J**) The migration ability of GC cell lines was determined by Transwell assay. Scale bars: 50 μm (*n* = 4). **P < 0.01, ***P < 0.001). Data are presented as the mean ± SD. One-way ANOVA with Tukey’s multiple-comparison test (**J**).

The CAP family functions by interacting with proteins. To explore the specific molecules to which CAP2 binds in promoting GC metastasis, we performed GST pull-down and coimmunoprecipitation (co-IP) along with liquid chromatography–mass spectrometry to identify the proteins interacting with CAP2. A total of 269 proteins were identified in the GST pull-down assay (Figure 4A) and 424 proteins in the co-IP assay (Figure 4B). Then we screened these proteins according to the following criteria: proteins that were specifically in the experimental group with molecular weights of about 35, 45, and 53 kDa; protein scores more than 500; and proteins related to tumor metastasis. Of these, 5 proteins that meet the above criteria are GAPDH, enolase 1, annexin A1, malate dehydrogenase 2, and receptor for activated C kinase 1 (RACK1) (Supplemental Figure 5A). A robust combination between exogenous GST-CAP2 and RACK1 was observed in the GST pull-down assay, and an endogenous CAP2/RACK1 complex was observed by co-IP, suggesting that CAP2 can interact with RACK1 (Figure 4, C and D). However, other proteins could not bind to CAP2. Immunofluorescence analysis showed that CAP2 and RACK1 were colocalized in MKN45 and BGC823 cell lines (Figure 4E and Supplemental Figure 5B). Collectively, our data indicate that RACK1 is a direct binding partner for CAP2.

RACK1 promotes cell migration by binding to focal adhesion kinase (FAK) and enhancing FAK and ERK1/2 activity (11, 12). Our results showed that CAP2 did not affect the RNA level of *FAK*/*MEK*/*ERK* but enhanced the phosphorylation of FAK and ERK (Figure 4F and Supplemental Figure 5, C and D). Knockdown of CAP2 downregulated the activity of FAK and ERK (Supplemental Figure 5E). Moreover, overexpression of CAP2 enhanced the binding of RACK1 to FAK (Figure 4, G and H). Cotransfection of CAP2 and RACK1 markedly enhanced both cell migration and invasion and FAK/ERK phosphorylation compared with CAP2 or RACK1 transfection alone (Figure 4, I and J, and Supplemental Figure 5, F and G). Moreover, the GEPIA and TNMplot databases showed that *RACK1* expression was significantly higher in GC tissues than in non-tumor tissues (Supplemental Figure 6, A and B). GEPIA and Kaplan-Meier Plotter revealed that patients with higher RACK1 expression had a poorer prognosis than those with lower RACK1 expression (Supplemental Figure 6, C and D). Gene set enrichment analysis showed that *CAP2* expression was positively related to focal adhesion and active MAPK signaling pathways (Supplemental Figure 6, E and F), consistent with our results. Taken together, our findings suggest that CAP2 enhances the activation of the FAK/MEK/ERK pathway by binding to RACK1.

### CAP2 competitively binds to domains WD5 to WD7 of RACK1 and dissociates SRC.

**Figure 5:**
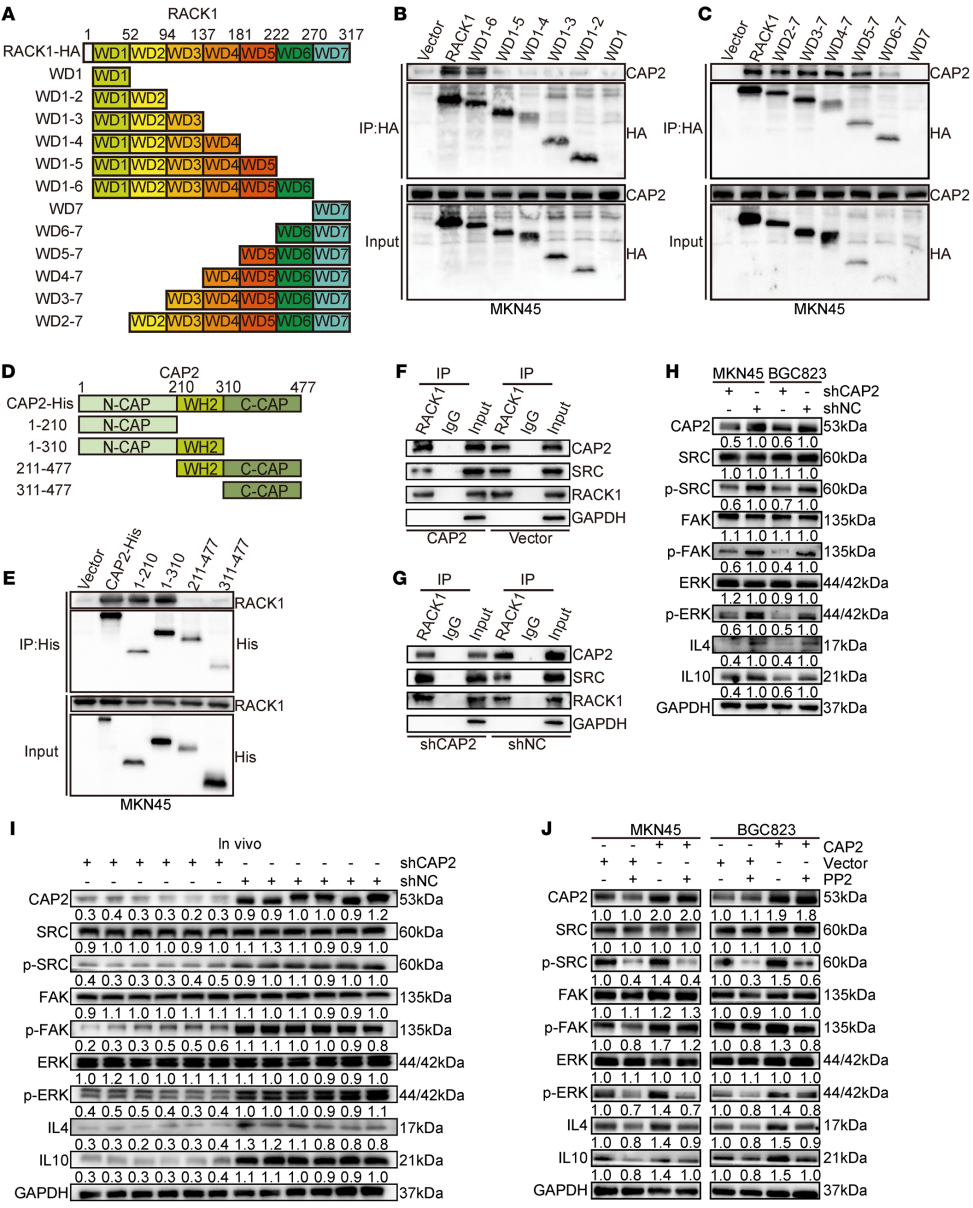
CAP2 competitively binds to domains WD5–WD7 of RACK1 and dissociates SRC. (**A**) Schematic representation of the RACK1 domains. (**B**) HA-tagged WT-RACK1, WD6-1, WD5-1, WD4-1, WD3-1, WD2-1, and WD1 were individually overexpressed in MKN45 cells, and anti-HA–tagged antibodies were used for co-IP. (**C**) HA-tagged WT-RACK1, WD2-7, WD3-7, WD4-7, WD5-7, WD6-7, and WD7 were individually overexpressed in MKN45 cells, and anti-HA–tagged antibodies were used for co-IP. (**D**) Schematic diagram of CAP2 protein truncation. (**E**) Overexpression of His-tagged WT-CAP2, CAP2(1–210bp), CAP2(1–310bp), CAP2(211–477bp), and CAP2(311–477bp) in MKN45 cells, immunoprecipitated with an anti-His-tag antibody. (**F** and **G**) Immunoprecipitation was performed using an IP-grade anti-RACK1 antibody. The level of SRC binding to RACK1 in GC cells with or without CAP2 expression was detected by Western blotting. (**H**) The expression and phosphorylation levels of the SRC/FAK/ERK signaling pathway in GC cells with or without CAP2 knockdown were detected by Western blotting. (**I**) Western blotting was conducted to determine the expression and phosphorylation levels of SRC/FAK/ERK/IL-4 and IL-10 in xenografted tumors. (**J**) SRC inhibitor (PP2) was added to GC cells overexpressing CAP2, and the expression and phosphorylation levels of SRC/FAK/ERK/IL-4 and IL-10 were detected by Western blotting.

RACK1 belongs to the WD repeat protein family and contains a 7-bladed propeller structure, which helps RACK1 act as a molecular scaffold with multiple binding sites. To elucidate the binding sites between CAP2 and RACK1, we generated 12 truncated RACK1 mutants to determine which domain of RACK1 interacts with CAP2 (Figure 5A). Co-IP assays showed that only the wild-type and WD5 to WD7 domains of RACK1 bound to CAP2 (Figure 5, B and C, and Supplemental Figure 7, A and B). Mutants that do not contain WD5, WD6, and WD7 domains lose the ability to interact with CAP2. Mammalian CAP2 protein contains 3 functional domains: the N-terminal domain (1–210 aa), the C-terminal domain (311–477 aa), and the middle proline-rich domain (211–310 aa). To identify the specific domain of CAP2 that binds to RACK1, we constructed a truncated protein of CAP2 (Figure 5D). The 3 CAP2 mutants were cotransfected into GC cells with HA-RACK1, and the cell lysates were coimmunoprecipitated with anti-HA or anti-His antibodies. The results showed that the N-terminal domain of CAP2, but neither the C-terminal domain nor the middle proline-rich domain, was able to immunoprecipitate RACK1 (Figure 5E and Supplemental Figure 7C), suggesting that the N-terminal domain of CAP2 directly binds to RACK1.

SRC activates the ERK pathway by promoting the phosphorylation of FAK. The WD6 domain of RACK1 binds to SRC and inhibits its tyrosine kinase activity (13–15). We speculate that CAP2 can competitively bind to the WD6 domain of RACK1 and release SRC. Co-IP showed that overexpression of *CAP2* weakened the binding ability between RACK1 and SRC. Conversely, the binding of RACK1 to SRC was enhanced after the knockdown of *CAP2* (Figure 5, F and G). Consistent with our hypothesis, overexpression of *CAP2* enhanced the phosphorylation of SRC, and knockdown of *CAP2* inhibited the phosphorylation of SRC (Figure 5H and Supplemental Figure 7D). Moreover, in xenograft tumors, the phosphorylation of SRC, FAK, and ERK was decreased in the shCAP2 group compared with the shNC group (Figure 5I). PP2, a specific SRC inhibitor, partially reversed activation of the FAK/ERK signaling pathway caused by *CAP2* overexpression (Figure 5J). In summary, on the one hand, CAP2 binds to RACK1 to promote RACK1-mediated FAK phosphorylation; on the other hand, CAP2 competitively binds to domains WD5 to WD7 of RACK1 and dissociates SRC to further promote the phosphorylation of FAK/ERK.

### GC tissues with high CAP2 expression are rich in M2 macrophages.

**Figure 6:**
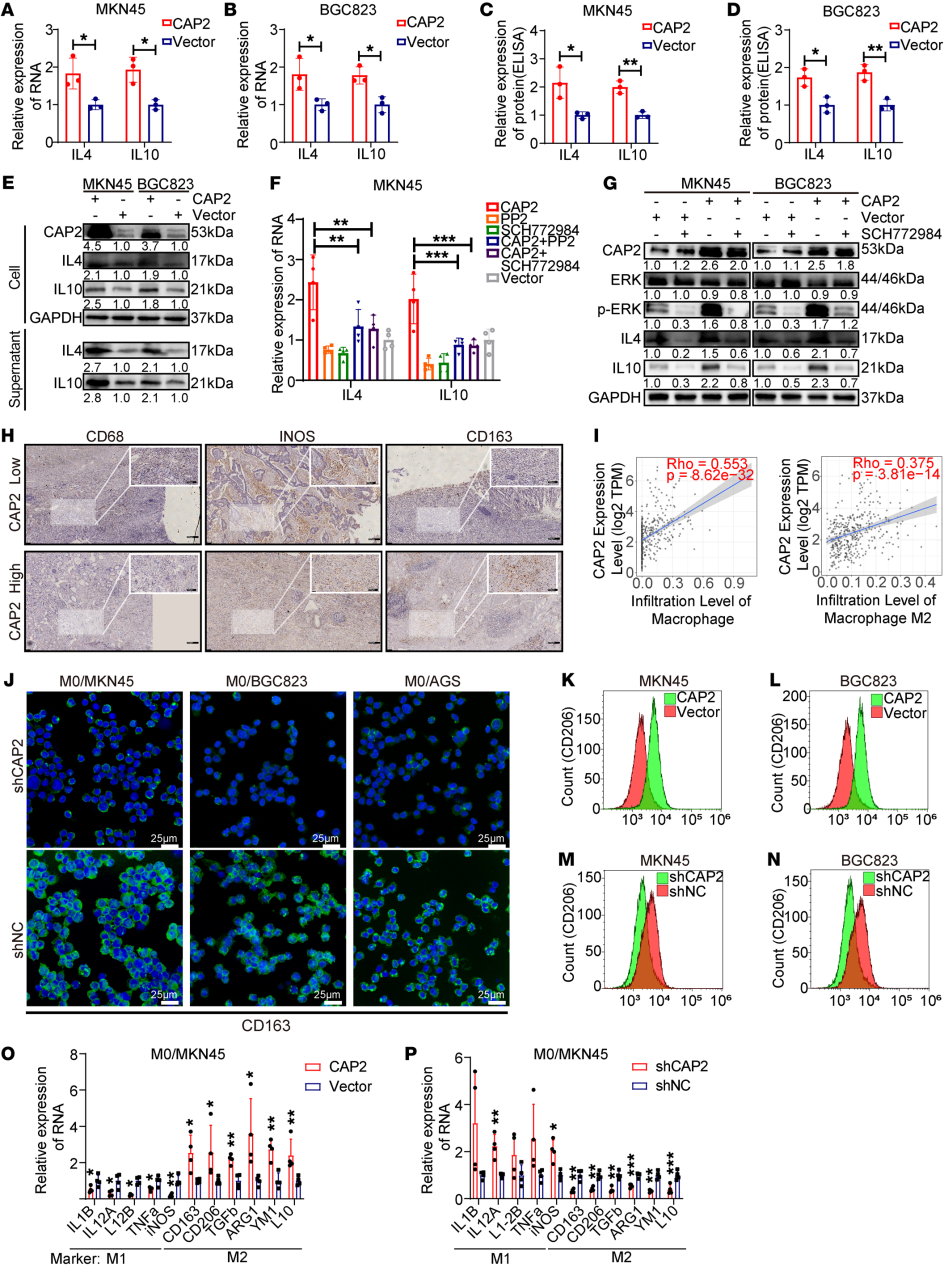
GC tissues with high expression of CAP2 are rich in M2 macrophages. (**A** and **B**) Effects of *CAP2* overexpression on the mRNA levels of *IL4* and *IL10* were assessed using RT-PCR (*n* = 3). (**C** and **D**) Expression levels of IL-4 and IL-10 in the supernatant of GC cells were detected by ELISA (*n* = 3). (**E**) GC cell supernatants were concentrated using ultrafiltration tubes and subjected to Western blotting. (**F**) SRC inhibitor (PP2) and ERK inhibitor (SCH772984) were added to MKN45 cells, and the RNA levels of *IL4* and *IL10* were detected by RT-PCR (*n* = 4). (**G**) ERK inhibitor (SCH772984) was added to GC cells, and the protein levels of IL-4 and IL-10 were detected by Western blotting. (**H**) Protein expression of CD68, iNOS, and CD163 in GC paraffin-embedded specimens was determined using IHC. Scale bars: 200 μm; 100 μm (insets). (**I**) Relationship between CAP2 and macrophage cell infiltration was analyzed using the TIMER2.0 website. (**J**) Expression of CD163 in macrophages was detected by immunofluorescence. Green staining indicates CD163 expression. Scale bars: 25 μm. (**K**–**N**) Expression of CD206 in macrophages was detected using flow cytometry. (**O** and **P**) Expression of markers for M1 and M2 macrophages after coculture of GC cells and macrophages (*n* = 4). Data are presented as the mean ± SD. Two-tailed unpaired Student’s *t* test (**A**–**D**, **O**, and **P**), 1-way ANOVA with Tukey’s multiple-comparison test (**F**). *P < 0.05, **P < 0.01, ***P < 0.001.

Phosphorylation of ERK1/2 promotes the expression of IL-4 and IL-10 (16). We speculated that CAP2 could enhance IL-4 and IL-10 by promoting the phosphorylation of ERK1/2. Our results showed that CAP2 was positively correlated with the mRNA expression of *IL4* and *IL10* (Figure 6, A and B, and Supplemental Figure 8, A–D) and promoted the secretion of IL-4 and IL-10 (Figure 6, C and D, and Supplemental Figure 8, E and F). Western blot analysis also showed that CAP2 promoted the expression and secretion of IL-4 and IL-10 proteins (Figure 5I, Figure 6E, and Supplemental Figure 8G). In addition, treatment with SCH772984, an inhibitor of ERK, inhibits the phosphorylation of ERK and reduces the expression of *IL4* and *IL10*. Moreover, SCH772984 reversed the increased phosphorylation of ERK1/2 and the expression of *IL4* and *IL10* caused by CAP2 (Figure 6, F and G, and Supplemental Figure 8H). These results suggest that CAP2 upregulates the expression of IL-4 and IL-10 by promoting ERK phosphorylation.

Tumor cells secrete cytokines, such as IL-4 and IL-10, which participate in the regulation of the phenotype of TAMs in the tumor microenvironment (17–20). To investigate the correlation between CAP2 and TAM infiltration in GC, we first examined the expression of inducible nitric oxide synthase (iNOS) (M1 marker), CD163 (M2 marker), and CD68 (macrophage marker) by immunohistochemistry. There were more CD163^+^ cells in GC with LNM than in GC without LNM, while the numbers of CD68^+^ cells were similar in both groups (Supplemental Figure 8I). In addition, the number of CD68^+^ cells in the high CAP2 expression group was similar to that in the low CAP2 expression group. More CD163^+^ cells were found in high CAP2 expression tumor tissues, while there were more iNOS^+^ cells in low CAP2 expression tumor tissues, suggesting that CAP2 promotes M2 polarization but does not affect the chemotaxis of macrophages (Figure 6H and Supplemental Figure 8J). A positive correlation between *CAP2* and *CD163*/*CD206* mRNA levels was observed in GC patients using the GEPIA database (Supplemental Figure 8, K and L). TIMER2.0 analysis also showed that *CAP2* was positively correlated with macrophage immune score in GC (Figure 6I and Supplemental Figure 8, M and N).

To evaluate whether CAP2 promotes M2 polarization in vitro, we stably overexpressed CAP2 or shCAP2 in GC cells and cocultured GC cells with THP1 human monocytes. Immunofluorescence analysis revealed that the knockdown of CAP2 significantly decreased the number of CD163^+^CD206^+^ macrophages (Figure 6J and Supplemental Figure 9, A–C). Flow cytometry revealed that the overexpression of *CAP2* in GC cells increased the polarization of CD163^+^CD206^+^ macrophages (Figure 6, K and L, and Supplemental Figure 9, D and E), whereas knockdown of *CAP2* decreased the polarization of CD163^+^CD206^+^ macrophages (Figure 6, M and N, and Supplemental Figure 9, F and G). Similar effects for macrophage markers were observed using quantitative real-time PCR (qPCR) (Figure 6, O and P, and Supplemental Figure 9, H and I). These results further indicate that CAP2 expression in GC cells promotes macrophage microenvironment differentiation into the M2-like phenotype.

### TAMs promote CAP2 expression through TGFB1-mediated activation of JUN.

**Figure 7:**
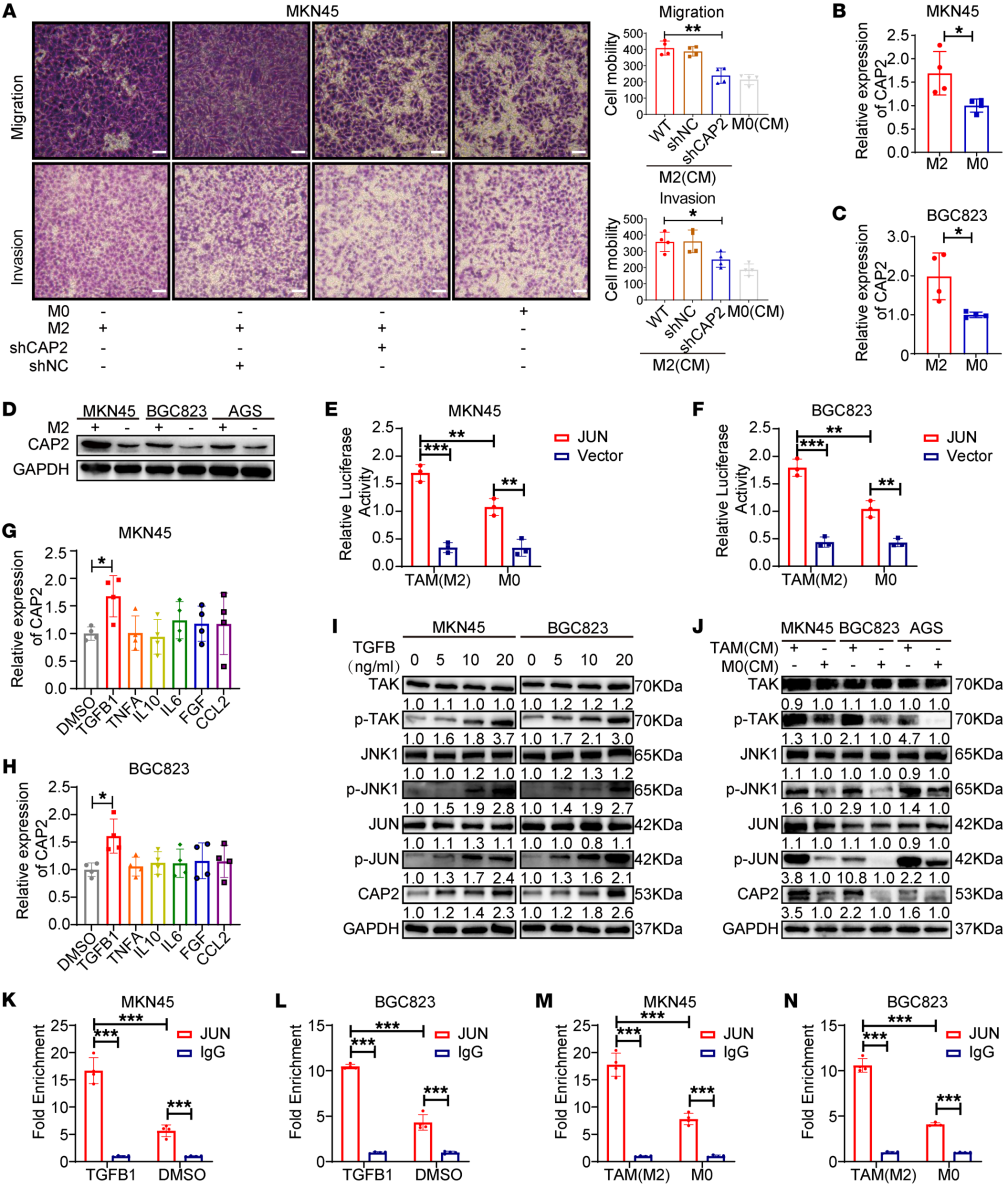
TAMs promote CAP2 expression through TGFB1-mediated activation of JUN. (**A**) The migration and invasion abilities of GC cells were determined by Transwell assay after the GC cells were induced by TAM-conditioned medium (*n* = 4). Scale bars: 50 μm. (**B** and **C**) Expression of CAP2 in GC cells induced by TAM-conditioned medium was detected by RT-PCR (*n* = 4). (**D**) Expression of CAP2 in GC cells induced by TAM-conditioned medium was detected by Western blotting. (**E** and **F**) Luciferase activity was detected after the GC cells were induced by TAM-conditioned medium (*n* = 3). (**G** and **H**) Expression of *CAP2* was detected by RT-PCR after the GC cells were treated with cytokines (*n* = 4). (**I**) Expression of TAK/JNK/JUN signaling pathway proteins was detected by Western blotting after the GC cells were treated with TGFB1. (**J**) Expression of TAK/JNK/JUN signaling pathway proteins was detected by Western blotting after the GC cells were induced by TAM-conditioned medium. (**K** and **L**) The binding ability of JUN to the *CAP2* promoter region was detected by ChIP after the GC cells were treated with TGFB1 (*n* = 4). (**M** and **N**) The binding ability of JUN to the *CAP2* promoter region was detected by ChIP after the GC cells were induced by TAM-conditioned medium (*n* = 4). Data are presented as the mean ± SD. One-way ANOVA with Tukey’s multiple-comparison test (**A**, **G**, and **H**), 2-tailed unpaired Student’s *t* test (**B** and **C**), 2-way ANOVA test (**E**, **F**, and **K**–**N**). *P < 0.05, **P < 0.01, ***P < 0.001.

An increasing amount of evidence shows that macrophages can promote the metastasis and progression of cancer cells (21, 22). To study the role of TAMs in GC metastasis, we established a coculture system of M2 macrophages and GC cells. Transwell assays showed that M2 macrophages enhanced the migration and invasion of MKN45 and BGC823 cell lines, and the knockdown of *CAP2* partially abolished this effect of M2, indicating that CAP2 is involved in the promotion of GC cell functions by regulating macrophages (Figure 7A and Supplemental Figure 10A). However, TAMs could promote GC cell proliferation, which was not affected by CAP2 in vitro (Supplemental Figure 10, B–E).

Interestingly, we found that TAMs promoted the mRNA and protein expression of CAP2 in GC cells (Figure 7, B–D). Given that JUN activates the transcription of *CAP2* and promotes CAP2 expression, we speculated that TAMs enhance *CAP2* mRNA expression by regulating JUN. The binding of JUN to the promoter region of *CAP2* was markedly increased in coculture with TAMs (Figure 7, E and F).

Cytokines are secreted by TAMs and promote GC progression (23). Therefore, we treated GC cells with cytokines and detected the expression of *CAP2*. TGFB1, rather than other cytokines, significantly enhanced the mRNA expression of *CAP2* (Figure 7, G and H). Evidence suggests that TGFB1 was able to activate JNK activity through a non-SMAD pathway and that JNK-mediated phosphorylation increases the transcriptional efficiency of JUN by enhancing its binding to gene promoters (24–26). Thus, we tested the effect of TGFB1 and TAMs on the TGFB1/TGFB1-activated kinase 1 (TAK1)/JNK pathway of GC cells. The results show that TGFB1 and TAMs can phosphorylate TAK1, JNK, and JUN in a cascade reaction (Figure 7, I and J), but had no effect on the RNA expression of *TAK1*, *JNK*, or *JUN* (Supplemental Figure 10, F and G). In addition, treatment of GC cells with TGFB1 or TAMs increased the binding of JUN to the *CAP2* promoter region (Figure 7, K–N, and Supplemental Figure 10, H and I). In addition, GEPIA showed that *CAP2* is positively correlated with *TAK1*, *JNK*, or *JUN* (Supplemental Figure 10J). Together, these results indicate that TAM activates TAK1/JNK/JUN signaling through secretion of TGFB1 to upregulate CAP2 expression.

### Salvianolic acid B is a putative molecular inhibitor of CAP2 to suppress GC progression.

**Figure 8:**
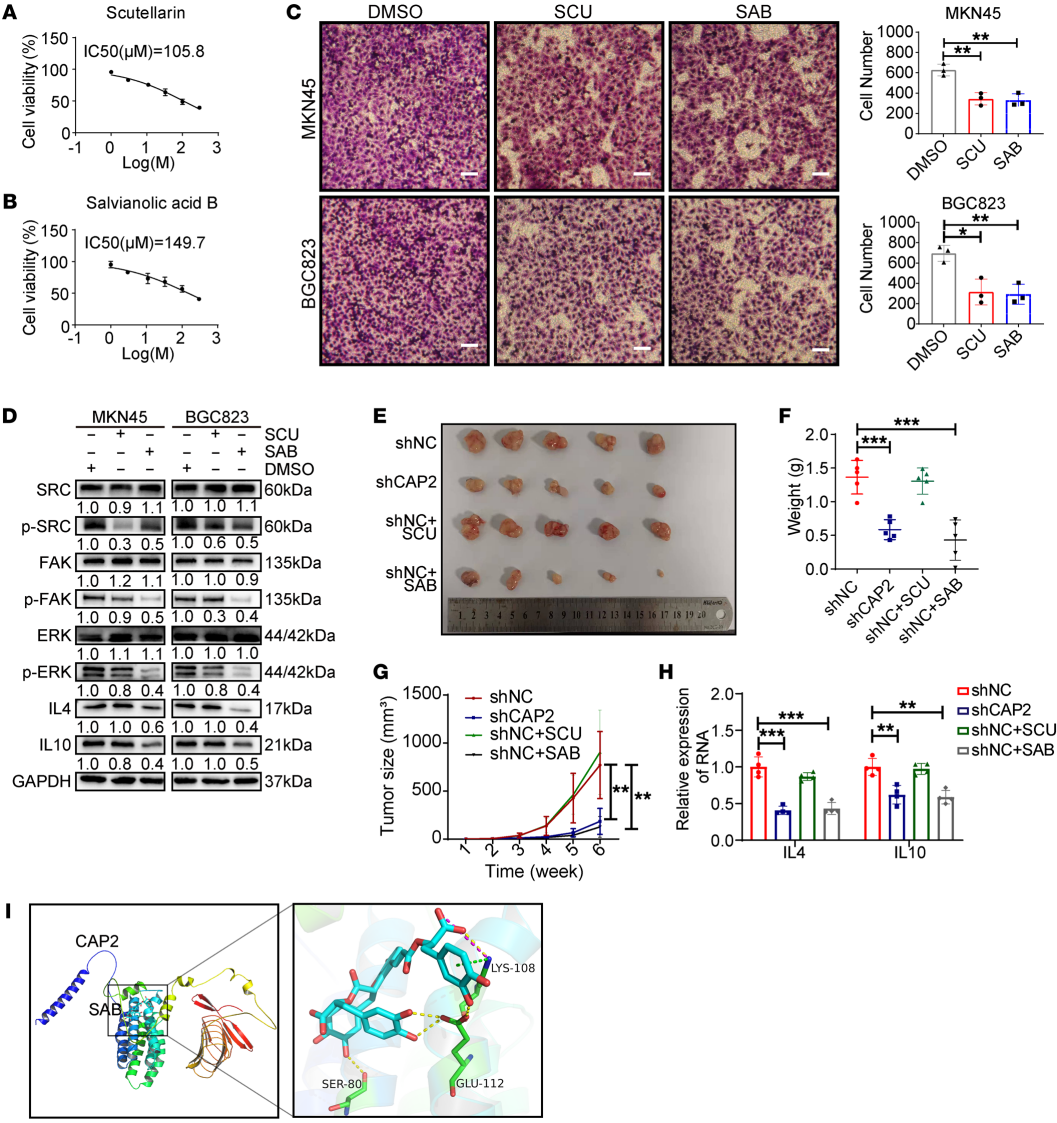
Salvianolic acid B is a putative molecular inhibitor of CAP2 and suppresses GC progression. (**A** and **B**) IC_50_ determination of scutellarin (SCU) and salvianolic acid B (SAB) on MKN45 cells. After treatment of GC cells with a series of doses (1, 3.5, 11, 33, 100, and 300 μM) of inhibitors for 48 hours, cell viability was determined by CCK-8 assay. (**C**) Transwell migration assay of GC cells treated with SCU and SAB. Scale bars: 50 μm. (**D**) Protein expression of SRC/FAK/ERK/IL-4/IL-10 was determined by Western blotting after the GC cells were treated with SCU and SAB. (**E** and **F**) Mice subcutaneously injected with LV-NC MKN45 cells were treated with SCU and SAB, and then xenograft tumors were extracted (**E**) and weighed (**F**). (**G**) The growth curves of xenograft tumors were plotted based on the tumor size. The tumor size (*V*) was calculated based on the equation *V* = (length × width^2^)/2 (*n* = 5). (**H**) RNA expression of *IL4* and *IL10* in tumors was determined by quantitative PCR (*n* = 4). (**I**) Autodock predicts molecular docking of CAP2 with salvianolic acid B. Data are presented as the mean ± SD. *P < 0.05, **P < 0.01, ***P < 0.001. One-way ANOVA with Tukey’s multiple-comparison test (**C**, **F**, and **H**), 2-way ANOVA test (**G**).

Given that CAP2 substantially promotes GC progression, we explored the potential molecular inhibitors of CAP2. We first simulated the structure of CAP2 protein with AlphaFold software (Supplemental Figure 11A) (https://alphafold.com/). Then we screened molecules binding to CAP2 in 7,507 compounds with Autodock software (https://autodock.scripps.edu/). A total of 20 compounds were selected for further study based on the lowest docking scores (Supplemental Table 1). To determine the antitumor effects of these compounds, we treated GC cells with these individual compounds and detected the viability of GC cells. Salvianolic acid A/B, scutellarin (SCU), plantamajoside, and proanthocyanidins had significant inhibitory effects on GC cells (Figure 8, A and B, and Supplemental Figure 11, B–K). The Transwell assay showed that SCU and salvianolic acid B (SAB) could significantly inhibit the migration ability of GC cells (Figure 8C and Supplemental Figure 11, L and M). In addition, SCU and SAB inhibited the phosphorylation of SRC/FAK/ERK (Figure 8D). These results suggest that SCU and SAB could serve as small-molecule inhibitors of CAP2 and have anticancer effects.

To explore whether SAB and SCU can be used as targets for GC therapy, SAB and SCU compounds were injected into tumor xenograft models. After 35 days of treatment, we found that compared with the control group, the mice treated with SAB had significantly reduced tumor volume (Figure 8, E and F), slower tumor growth rate (Figure 8G), and no obvious invasion (Supplemental Figure 11N), which was similar to mice in the shCAP2 group. However, SCU-treated mice showed no significant changes compared with the control group (Figure 8, E–G, and Supplemental Figure 11N). We dissected tumors from mice, isolated TAMs from tumors by magnetic beads, and detected macrophages with macrophage markers. Compared with the control group, M2-type macrophage infiltration was decreased in the shCAP2 group and the SAB group, but not in the SCU group (Supplemental Figure 11O). In addition, IL-4 and IL-10 expression was also decreased (Figure 8, D and H). These results indicate that SAB inhibits tumor growth and M2 cell polarization in vivo. In addition, we monitored SAB-treated mice for tumor lung metastasis. The results indicated that 7 days after tumor inoculation, lung metastases were not evident in either group. Fourteen days after tumor inoculation, the number of lung metastases in the SAB-treated group was lower than that in the control group. Forty-two days after tumor inoculation, the number and volume of lung metastases in the SAB-treated group were significantly lower than those in the control group (Supplemental Figure 12, A and B). Furthermore, we monitored the survival status of the mice. The results indicated that the survival time of the mice in the SAB-treated group was significantly prolonged, and by the end of the experiment, 6 mice in the SAB-treated group were still alive, whereas only 1 mouse in the control group was alive (Supplemental Figure 12C). The above results prove that SAB can inhibit the metastasis of GC in vivo and prolong the survival of mice. To further clarify the role of the inhibitor, we performed virtual docking through Autodock software, and the results showed that SAB mainly bound to the S80, K108, and E112 amino acid residues of the N terminal of CAP2 (Figure 8I and Supplemental Figure 13), suggesting that SAB may act by inhibiting the N-terminal domain of CAP2. In summary, SAB as a small-molecule inhibitor of CAP2 could suppress the growth and metastasis of GC.

## Discussion

Accumulating evidence has revealed that CAP2 plays a role in the progression of many cancers (27–29). However, the functional roles of CAP2 in GC metastasis remain largely unknown. In this study, we first identified that the *CAP2* expression level was upregulated stepwise in nontumorous gastric mucosa, nonmetastatic GCs, and metastatic GCs. High expression of CAP2 is associated with high LNM and GC clinical stage. Moreover, survival analysis showed that patients with high CAP2 expression had a shorter survival than those with low CAP2 expression. Next, to further study the mechanism of high expression of CAP2 in GC, we defined the proximal promoter of *CAP2* and confirmed that JUN as a transcription factor upregulates CAP2 in GC. JUN is a basic leucine zipper transcription factor and regulates gene transcription as a homodimer or heterodimer. JUN is the most widely studied protein in the activator protein-1 complex and is involved in many cellular activities such as proliferation, apoptosis, tumorigenesis, and metastasis (30, 31). In this study, we found that overexpression of *CAP2* in GC is caused by the transcription factor JUN binding to the *CAP2* core promoter region and activating its transcription. JUN can cause the migration and infiltration of tumor cells, consistent with the function of CAP2 in GC cells (32, 33).

To further understand the function of CAP2, we conducted a series of experiments and found that CAP2 promotes the migration and invasion of GC cells in vitro and in vivo, consistent with the high expression of CAP2 in GC with LNM. At present, the underlying molecular mechanisms of CAP2 in GC progression remain unknown. We demonstrated that CAP2 is directly bound to RACK1. RACK1 has seven β-propeller blades (WD1–WD7), which can serve as binding sites for multiple interaction partners, enabling it to act as a scaffold protein (34–36). RACK1 is bound to FAK and regulates its activity (37). Our results identify that CAP2 can bind to the WD5–WD7 domains of RACK1 and enhance the phosphorylation of FAK. SRC interacts with the sixth WD repeat in RACK1, and the tyrosine kinase activity is inhibited (13, 38). Therefore, we speculate that CAP2 competes with SRC to bind to the same region in RACK1, causing SRC to be released from RACK1 and be phosphorylated. The combination of phosphorylated FAK (Tyr397) and active SRC (Tyr416) further phosphorylates Tyr925 of FAK to induce the complete activation of FAK (14, 15). FAK in the active state acts as the initiation factor of the ERK pathway to activate ERK phosphorylation (39). ERKs are a broadly conserved family of serine/threonine protein kinases that are involved in many cellular processes, and they regulate the transcription of multiple target genes (40, 41).

It has been reported that phosphorylated ERK1/2 activates the transcription of *IL4* and *IL10* and promotes the expression of IL-4 and IL-10 (16). The research of Ahad et al. shows that ERK inhibitors reduce IL-4 expression and secretion (42). Tripathi et al. found that ERK1 and ERK2 proteins were directly recruited at the proximal promoter of the *IL4* gene and observed the consequent initiation of *IL4* gene transcription (43). In addition, activation of ERK regulates IL-10 expression, and IL-10 production decreases in the presence of chemical inhibitors of ERK or ERK-deficient cells. Highly selective synthesis inhibitors of ERK1/2 significantly inhibit IL-10 expression in DCs (44). Inhibition of the ERK and p38 pathways in macrophages leads to the almost complete elimination of IL-10 production. ERK activation causes rapid and transient phosphorylation of histone H3 at specific regions of the *IL10* promoter, resulting in a transient exposure of the *IL10* promoter to the transcription factors that bind there, which in turn activates the transcription of *IL10* (45). Tumor cells secrete a variety of cytokines, which can participate in the regulation of the phenotype of TAMs in the tumor microenvironment. IL-4, IL-10, and IL-13, among others, could polarize macrophages toward the M2 phenotype (19, 46). Most TAMs exhibit the M2 phenotype. M2-like TAMs are positive regulators of tumor growth and metastasis, whereas M1-like TAMs have the opposite effects (20, 47). In this study, we examined the role of CAP2 in the production of cytokines in GC cells. We found that the expression and secretion of IL-4 and IL-10 in GC cells are positively correlated with the expression of CAP2. ERK inhibitors could reverse CAP2-mediated increased expression of IL-4 and IL-10. We demonstrate that CAP2 expression in GC cells promotes M2-like polarization of TAMs. Moreover, macrophages cocultured with GC cells with high CAP2 expression substantially promote cell migration and invasion. The numbers of M2 TAMs are positively correlated with the expression level of CAP2 in GC tissues. Our results suggest that CAP2 increases the expression and secretion of IL-4 and IL-10 in GCs by enhancing ERK phosphorylation, thereby promoting the polarization of M2 macrophages.

Interestingly, we also found that TAMs (M2) can promote the expression of CAP2 in GC cells. The results showed that M2 macrophages secreted a large amount of TGFB1 and bound to the GC cell membrane receptor TGFB1R to activate the TAK1/JNK/JUN pathway. TGFB1 belongs to a TGFB1 superfamily group that regulates cell growth and differentiation. The combination of TGFB1 ligand and TGFB1 receptor promotes the formation of a heterotetrameric receptor complex composed of TGFB1RI and TGFB1RII (25). The receptor complex can phosphorylate and activate TAK1 and JNK (48, 49). JNK1 binds to the JUN transactivation domain and phosphorylates to activate JUN (50). Phosphorylation of JUN can increase its binding ability to target gene promoters, thereby enhancing its transcriptional activity (51, 52). Therefore, TAMs activate the TAK1/JNK/JUN pathway in GC cells by secreting TGFB1, thereby upregulating CAP2 expression. Together, these data demonstrate a close crosstalk between TAMs and tumor cells. Previous studies have shown that M2-like TAMs can accelerate tumor growth and promote tumor invasion and metastasis (53). Therefore, targeting TAMs has become one of the main strategies in current tumor immunotherapy research. Despite some success with these treatments, their effectiveness remains limited. In our study, the expression of CAP2 in GC forms a mutually reinforcing positive-feedback loop with the polarization of TAMs. Breaking this cycle may provide new therapeutic strategies for the treatment of tumor metastasis.

Currently, the antitumor role of natural small-molecule compounds has garnered increasing attention. However, there are still no inhibitors targeting CAP2 in clinical practice. Here, we screened and found that SAB could be a small-molecule compound against CAP2. SAB is the main bioactive constituent of salvia. It has been reported to have anticancer, antiinflammatory, and cardioprotective effects. Currently, salvianolic acid is considered as an effective anticancer molecule (54). In addition, SAB is a safe natural compound with no observed toxicity to the heart, brain, kidney, lung, and liver at various doses, and it has an LD_50_ value of 1,747 mg/kg, which is 100 times its effective dose (55). Our study confirmed that SAB has antitumor activity and can inhibit the SRC/FAK signaling pathway in vitro and in vivo. In addition, SAB could inhibit the metastasis of GC cells in vivo and prolong the survival of mice. The molecular docking revealed that SAB targets the N-terminal domain of CAP2. These data provide evidence for the clinical application of SAB to target CAP2 and inhibit GC progression.

In conclusion, high expression of CAP2 promotes the metastasis of GC cells in vitro and in vivo and causes macrophages to polarize to TAMs (M2 phenotype). Meanwhile, polarized TAMs further promoted the transcription of CAP2 in GC cells by secreting TGFB1. A positive-feedback loop is formed between CAP2 and TAMs to jointly promote the metastasis of GC. In summary, CAP2 is a potential prognostic factor that has a promotive effect on the metastasis of GC cells and the polarization of M2 macrophages. Together, these data provide a better understanding of the mechanisms of tumor-macrophage interaction, and targeting CAP2 could provide new ideas for the treatment of GC metastasis.

## Methods

Further information can be found in Supplemental Methods.

### Human GC samples.

Fresh GC tissues were obtained from the Qilu Hospital of Shandong University from 2010 to 2014. Moreover, from 2004 to 2010, 124 paraffin specimens of GC were collected from the Qilu Hospital of Shandong University for immunohistochemical examination.

### Cell culture and reagents.

Human GC cells (MKN45, BGC823, AGS, and HGC27), human gastric epithelial cell line GES-1, human monocytic leukemia cells (THP1), and human embryonic kidney 293T (HEK293T) cells were purchased from the Shanghai Cancer Institute (Shanghai, China). The above cells were cultured in RPMI 1640 (Boehringer Ingelheim, Herzliya Pituach, Israel) medium containing 10% FBS (Boehringer Ingelheim).

### siRNA transfection.

Sequences of siRNAs targeting the human gene *CAP2* were referred to in the literature (Supplemental Table 2). siRNAs (GenePharma) were synthesized and transfected into GC cell lines using X-tremeGENE (Roche Applied Science). In brief, 1 × 10^5^ cells were seeded into a 12-well plate. After 12 hours, a mixture of siRNA or negative control and X-tremeGENE transfection reagent was added to each well. Transfection efficiency was monitored by quantitative PCR (qPCR).

### Transwell migration and invasion assays.

Transwell assays were performed as previously described (56).

### RNA extraction and qPCR.

Total RNA was extracted from GC cells, GC tissues, or macrophages using TRIzol reagent (Thermo Fisher Scientific). RNA was reverse-transcribed into cDNA using a reverse transcription kit (Toyobo) according to the manufacturer’s instructions. Next, qPCR was performed in a Bio-Rad CFXTM 96 C1000 real-time system using 5 μL SYBR Green Mix (Roche Diagnostic GmbH) plus 1 μg cDNA plus water. Primers were designed and produced by GenePharma (Supplemental Table 3).

### Immunohistochemistry.

Immunohistochemical (IHC) experiments were conducted with an SP9000 IHC kit according to the manufacturer’s instructions (ZSGB Bio). GC tissues were treated with anti-CAP2 (1:150; 15865-1-AP, Proteintech), anti-CD163 (1:200; ab182422, Abcam), anti-iNOS (1:200; ab283655, Abcam), and anti-CD68 (1:200; ab283654, Abcam) at 4°C for 12 hours. The IHC staining intensity was scored as follows: 0, negative staining; 1, light brown; 2, brown; and 3, dark brown. The stained area was scored as follows: 0, less than 5%; 1, 5%–25%; 2, 26%–50%; 3, 51%–75%; and 4, 76%–100%. The product of intensity and percentage was considered the final score. ROC curve analysis was used to obtain the cutoff values. A final score greater than 7 was defined as high expression of CAP2.

### Western blot assay.

After the proteins were resolved by SDS-PAGE, they were electrotransferred to PVDF membranes. Then, the membranes were incubated with primary antibodies at 4°C overnight. The primary antibodies used are shown in Supplemental Table 4. After washing, the membranes were incubated with goat anti-rabbit (1:5,000; 7074, Cell Signaling Technology [CST]) or anti-mouse secondary antibody (1:5,000; 7076, CST) for 1 hour at room temperature. Protein bands were visualized using supersensitive ECL chemiluminescent solution (6883, CST) and a MyECL Imager (QinXiang).

### Dual-luciferase reporter assay.

GC cells were plated in 24-well plates, and 12 hours later, the *CAP2* promoter or mutant luciferase vector was transfected into the cells using a transfection reagent (TurboFect, Thermo Fisher Scientific). After 48 hours, the cells were analyzed for luciferase activity using the Dual-Luciferase Reporter Assay System (Promega) according to the manufacturer’s instructions.

### ELISA.

Conditioned medium (CM) from GC cells or macrophages were used for the sandwich ELISA, according to the manufacturer’s instructions (MultiSciences Biotech Co.).

### Flow cytometry.

THP1 cells cocultured with GC cells were collected by centrifugation at 800*g* for 5 minutes and rinsed with cell staining buffer. Then cells were incubated with an anti-CD206 antibody (1:200; 321106, BioLegend) in the dark for 30 minutes. Next, the stained cells were washed and analyzed by flow cytometry.

### ChIP assay.

The nuclear DNA of GC cells was taken and immunoprecipitated with anti-JUN (sc-7345, Santa Cruz Biotechnology) or IgG antibody. Chromatin immunoprecipitation (ChIP) experiments were performed using the Chromatin Immunoprecipitation Kit (Merck) following the manufacturer’s instructions. The precipitated DNA was quantified using qPCR.

### Invasion and metastasis assays in vivo.

MKN45 cells were transfected with a LV-shRNA-CAP2 or LV-NC tagged with a green fluorescent protein. For the invasion assay, 4 × 10^5^ MKN45 cells were inoculated into the left axilla of 4-week-old male BALB/c nude mice (Vital River). For the metastasis, 4 × 10^5^ MKN45 cells were injected into the tail vein of mice. In addition, a group of LV-NC mice was subjected to 66 μg CAP2 siRNA (GenePharma) 3 times a week. Tumor volume was measured every 3 days. Furthermore, 5 weeks after implantation, the mice were sacrificed. The tumors, lungs, livers, and kidneys of mice were separated for sectioning and H&E staining. In vivo imaging of mice was visualized using the Carestream Molecular Imaging System (Carestream Health Inc.).

### GST pull-down assay.

GST tag and GST-CAP2 fusion proteins were purchased from Genecreate, and the GST pull-down assay was conducted according to the manufacturer’s instructions (catalog 21516, Thermo Fisher Scientific). Pulled down proteins were separated by gel electrophoresis for silver nitrate staining and Western blotting.

### Statistics.

All statistical analyses were conducted using SPSS 18.0 and GraphPad Prism 9 (GraphPad Software Inc.). The χ^2^ test was used to assess the relationship between CAP2 expression and clinicopathological parameters. Two-tailed unpaired Student’s *t* test (2 experimental groups) or 1-way ANOVA with multiple comparisons (more than 2 experimental groups) was used for continuous data, as appropriate. Kaplan-Meier method was applied to plot survival curves and analyzed with the log-rank test. *P* less than 0.05 was considered statistically significant.

### Study approval.

All patients gave written informed consent. All animal experiments were performed under a protocol approved by the Ethics Committee of the School of Basic Medical Sciences of Shandong University.

### Data availability.

Survival analyses are available online from the GEPIA data set and Kaplan-Meier Plotter. Immune cell infiltration is available online from TIMER2.0. See complete unedited blots in the supplemental material. Values for all data points in graphs are reported in the Supporting Data Values file.

## Author contributions

PG contributed to design of the work and interpretation of data. GZ contributed to design of the work, performance of experiments, interpretation of data, and writing of the paper. XG, XW, RM, CL, ZG, and ZT contributed to performance of experiments. PZ and RZ contributed to interpretation of data.

## Supplementary Material

Supplemental data

Supporting data values

## Figures and Tables

**Table 1:**
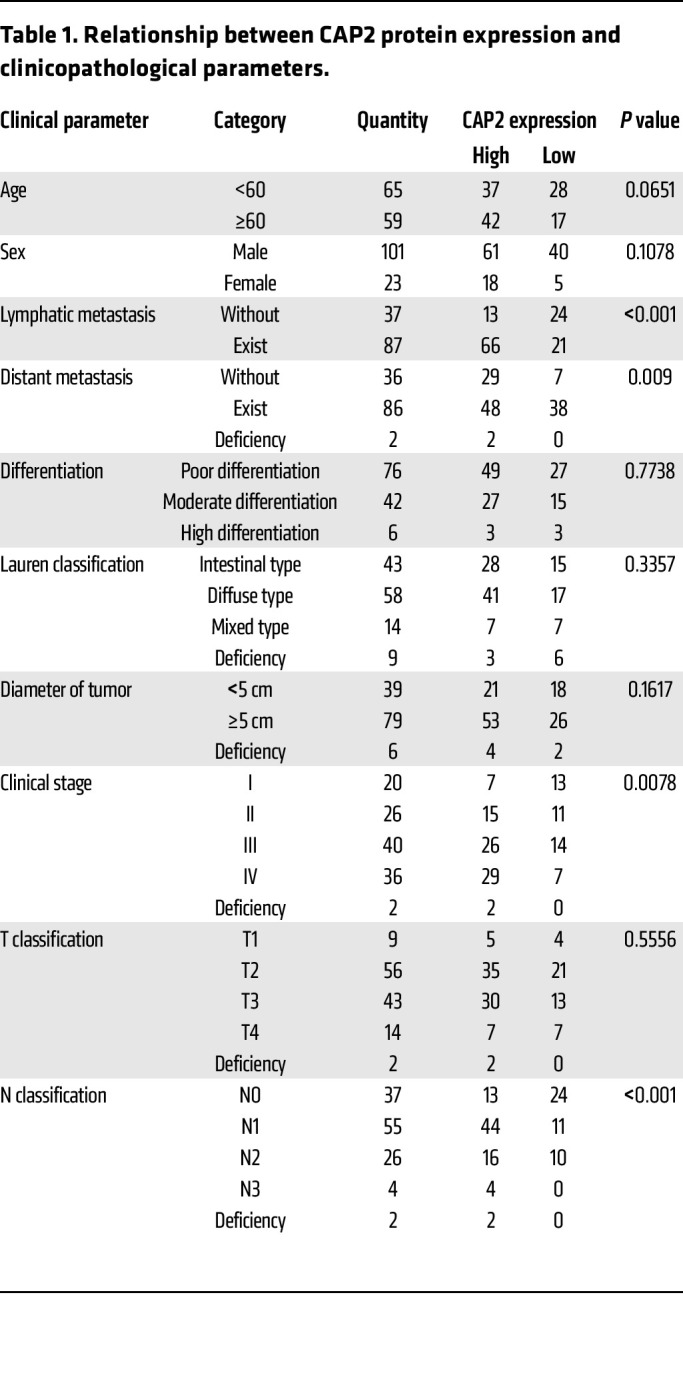
Relationship between CAP2 protein expression and clinicopathological parameters.

## Version 1 09/14/2023

In-Press Preview

## Version 2 11/01/2023

Electronic publication
