# Supplementary material for: CAP2 promotes gastric cancer metastasis by mediating the interaction between tumor cells and tumor-associated macrophages
Source: J Clin Invest. 2023 Nov 1;133(21):e166224. doi: 10.1172/JCI166224 (PMC10617780; doi:10.1172/JCI166224)
Supplement: Supplemental data [file jci-133-166224-s229.pdf]

CAP2 promotes gastric cancer metastasis by mediating the interaction between tumor cells and tumor-associated macrophages

Guohao Zhang, Zhaoxin Gao, Xiangyu Guo, Ranran Ma, Xiaojie Wang, Pan Zhou, Chunlan Li, Zhiyuan Tang, Ruinan Zhao, Peng Gao

Supplementary material

Supplemental Table 1. Small molecule compounds

Name	Formula	MolWeight	docking score
Rosavin	C20H28O10	428.430	-9.008
Scutellarin	C21H18O12	462.360	-8.943
Oroxin B	C27H30O15	594.518	-8.210
Homoplantaginin	C22H22O11	462.403	-7.949
Harpagoside	C24H30O11	494.488	-7.881
Salvianolic acid B	C36H30O16	718.614	-7.539
Salvianolic acid A	C26H22O10	494.447	-7.487
Eriocitrin	C27H32O15	596.534	-7.388
Proanthocyanidins	C30H26O13	594.519640	-7.232
Corilagin	C27H22O18	634.452780	-7.365
Vaccarin	C32H38O19	726.632720	-7.013
Specnuezhenide	C31H42O17	686.655	-6.996
Plantamajoside	C29H36O16	640.587	-6.950
Dinoprost tromethamine	C24H45NO8	475.616	-6.946
Acarbose	C25H43NO18	645.604820	-6.926
Fluvastatin sodium (Lescol)	C24H25FNNaO4	433.448	-6.923
LDC000067	C18H18N4O3S	370.426	-6.916
Rosuvastatin Calcium	C44H54CaF2N6O12S2	1001.137	-6.916
Oroxin A	C21H20O10	432.377	-6.903
Forsythoside A	C29H36O15	624.587	-6.875

Supplemental Table 2. siRNA (mix) sequences targeting CAP2.

siCAP2-1	GGAGUGAACUUCAAGCAUA
siCAP2-2	GGAGUUGGAAGGAAAGAAA

Supplemental Table 3. The sequence of primers for qRT-PCR.

Gene	Forward primer	Reverse primer
CAP2(human)	5'-CCTGCCCTTGGATGGATAGC-3'	5'-GTGTGGTGTTCCTTGATGTATGC-3'
RACK1(human)	5'-GCTGGTCAAGGTATGGAACC-3'	5'-GCGTGTAAAGGTGTTTGCCT-3'
FAK(human)	5'-TTACCTGGGGAACCCCGACC-3'	5'-TGGTGAAGGATGAGGGCTCGT-3'
ERK1(human)	5'-GCTGAATCACATCCTGGGTAT-3'	5'-AGATCTGTATCCTGGCTGGAA-3'
MEK(human)	5'-GGGCTTCTATGGTGCGTTCTA-3'	5'-CCCACGGGAGTTGACTAGGAT-3'
JUN(human)	5'-GAGCTGGAGCGCCTGATAAT-3'	5'-CCCTCCTGCTCATCTGTCAC-3'
IL4(human)	5'-CTGCTTCCCCCTCTGTTCTTC-3'	5'-TGATATCGCACTTGTGTCCGTG-3'
IL10(human)	5'-CTGAGAACCAAGACCCAGACA-3'	5'-AAAGGCATTCTTCACCTGCTCC-3'
IL1B(human)	5'-AGCTGGAGAGTGTAGATCCCAA-3'	5'-ACGGGCATGTTTTCTGCTTG-3'
IL12A(human)	5'-CTCCTGGACCACCTCAGTTTG-3'	5'-AGGTTTTGGGAGTGGTGAAGG-3'
IL12B(human)	5'-CACAAAGGAGGCGAGGTTCT-3'	5'-TTTGGGTTCTTTCTGGTCCTT-3'
iNOS2(human)	5'-CGTGGAGACGGGAAAGAAGT-3'	5'-GACCCCAGGCAAGATTTGGA-3'
TNFA(human)	5'-GAGGCCAAGCCCTGGTATG-3'	5'-CGGGCCGATTGATCTCAGC-3'
CD163(human)	5'-GGGCTAATTCCAGTGCAGGT-3'	5'-GCTGACTCATTCCCACGACA-3'
CD206(human)	5'-GATTGCAGGGGGCTTATGGG-3'	5'-CGGACATTTGGGTTCGGGAG-3'
YM1(human)	5'-TGGGAGTGGAATGATGTGACG-3'	5'-TCTTCAGGTTGGGGTTCCTGT-3'
TGFB1(human)	5'-GCAACAATTCCTGGCGATACC-3'	5'-ATTTCCCCTCCACGGCTCAA-3'
ARG1(human)	5′- TGACGGACTGGACCCATCTT-3′	5′- GGCTTGTGATTACCCTCCCG--3′
TAK(human)	5'-GCCCTCAACCACGACTTCTT-3'	5'-GGATTGCGACTCTGGTTGGT-3'
JNK1(human)	5'-GATGCTGTGTGGAATCAAGCA-3'	5'-CCTCGGGTGCTCTGTAGTAG-3'

GAPDH(human)	5′-ACAACTTTGGTATCGTGGAAGG-3′	5′-GCCATCACGCCACAGTTTC-3′
Cd11B(mouse)	CATGAATGATGCTTACCTGGGTTATG	CCCAAAATAAGAGCCAATCTGG
Il12a(mouse)	5′-CTCAGTTTGGCCAGGGTCAT-3′	5′-TCTTCAGCAGGTTTCGGGAC-3′
Tnfα(mouse)	5′- CCTCTCATGCACCACCATCA-3′	5′- GCATTGCACCTCAGGGAAGA-3′
Il1b(mouse)	5′- AAGGGGACATTAGGCAGCAC-3'	5′- ATGAAAGACCTCAGTGCGGG-3'
Cd163(mouse)	5′-GCAAAAACTGGCAGTGGG-3′	5′-GTCAAAATCACAGACGGAG
Arg1(mouse)	5′-CAATGAAGAGCTGGCTGGTGT-3′	5′-GTGTGAGCATCCACCCAAATG
Tgfb(mouse)	5′-GTCCAAACTAAGGCTCGCCA-3′	5′-ATAGATGGCGTTGTTGCGGT-3′
Ym1(mouse)	5′-GAAGCTCTCCAGAAGCAATCCT-3′	5′-CTGGTAGGAAGATCCCAGCTGTA-3′
CAP2-ChIP(human)	5'-AGCCAAGTGGGTTTGGTTACT-3'	5'-CTGGGAGGAGAAGGAGCTAGA-3'

Supplemental Table 4. Antibodies

Antibody	Source	Dilution	Catalog Number
CAP2(WB)	Proteintech	1:1000	15865-1-AP
RACK1(WB)	Proteintech	1:1000	27592-1-AP
GAPDH(WB)	CST	1:1000	5174T
FAK(WB)	CST	1:1000	3285T
p-FAK(WB)	CST	1:1000	8556T
ERK1/2(WB)	CST	1:1000	4695T
p-ERK1/2(WB)	CST	1:1000	4370T
MEK(WB)	CST	1:1000	4694T
p-MEK(WB)	CST	1:1000	3958T
SRC(WB)	Santa Cruz	1:1000	sc-376476
p-SRC(WB)	CST	1:1000	6943T
TAK(WB)	CST	1:1000	5206S
p-TAK(WB)	CST	1:1000	9339S
JNK1(WB)	CST	1:1000	3708T
p-JNK1(WB)	CST	1:1000	9255S
JUN(WB)	Santa Cruz	1:1000	sc-7345
p-JUN(WB)	Abcam	1:1000	ab32385
IL4(WB)	CST	1:1000	12227S
IL10(WB)	CST	1:1000	12163S
His tag (WB)	CST	1:1000	3724S

HA tag (WB)	CST	1:1000	12698S
CAP2(IF)	Proteintech	1:100	15865-1-AP
RACK1(IF)	Proteintech	1:100	66940
CD163(IF)	Proteintech	1:100	16646S
CD206(IF)	Santa Cruz	1:100	sc-376108

CST, Cell Signaling Technology ; WB, western blot; IF, Immunofluorescence.

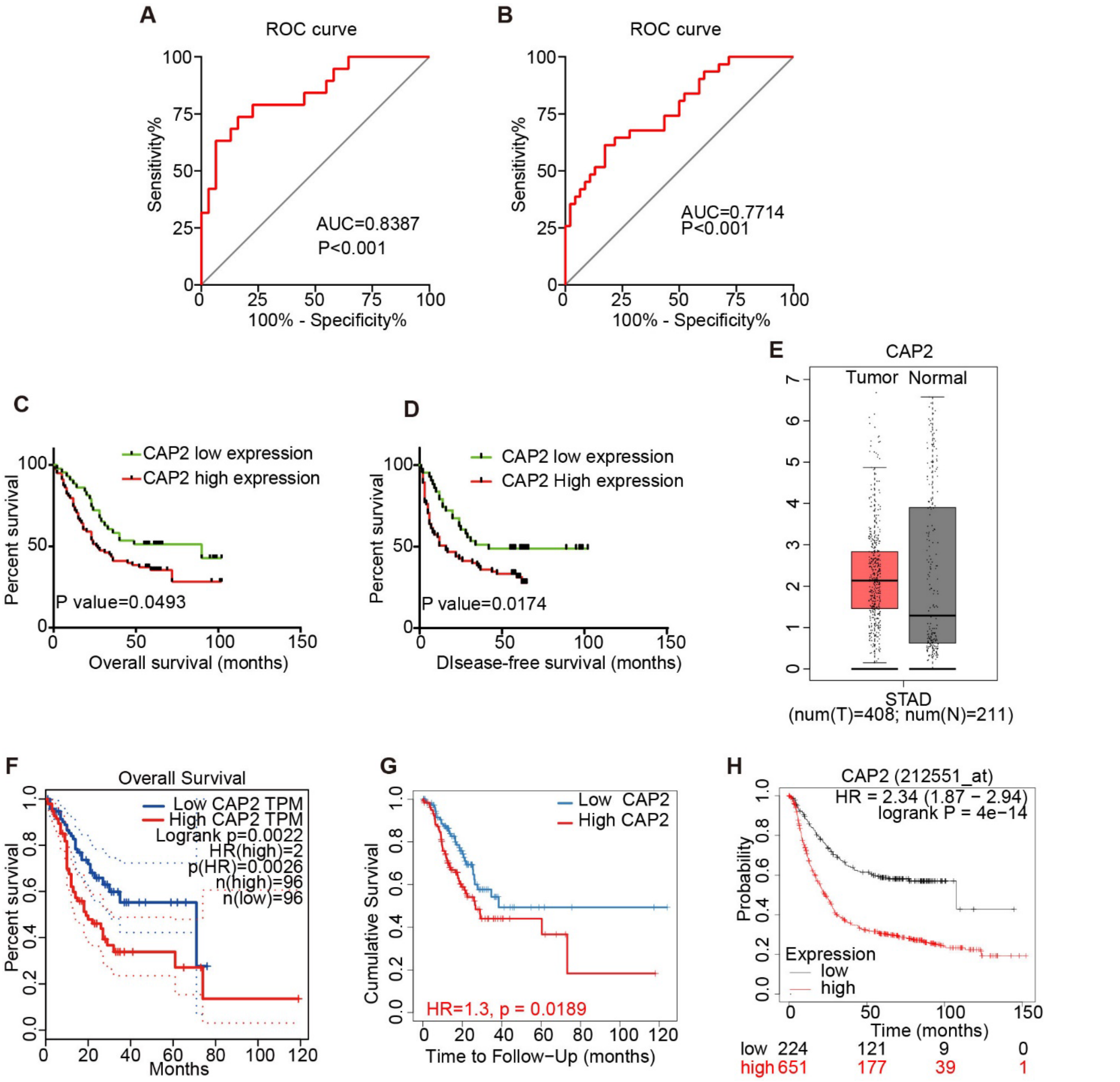

Supplemental Figure 1. CAP2 could be used as a diagnostic marker in GC. (**A**) Area under the ROC curve for *CAP2* in the differential diagnosis of gastric mucosa and GC. (**B**) Area under the ROC curve for *CAP2* in GC tissues with LNM or without LNM. (**C and D**) Kaplan–Meier curves of overall survival and disease-free survival for CAP2 expression (ROC curve analysis was used to obtain the cut-off values. The immunohistochemistry score of >7 was defined as high expression of CAP2. *n*[low] = 43, *n*[high] = 79). (**E**) The expression of *CAP2* in GC tissues and normal tissues was analyzed with GEPIA. (**F**) Correlation between CAP2 expression and overall survival of patients with GC in the GEPIA dataset (http://gepia.cancer-pku.cn/). (**G**) Timer2.0 online analysis showed that patients with high CAP2 expression had a poor prognosis compared with patients with low expression. (**H**) The Kaplan-Meier Plotter showed poor overall survival in patients with high CAP2 expression. logrank test (C, D). *$P < 0.05$, **$P < 0.01$, ***$P < 0.001$.

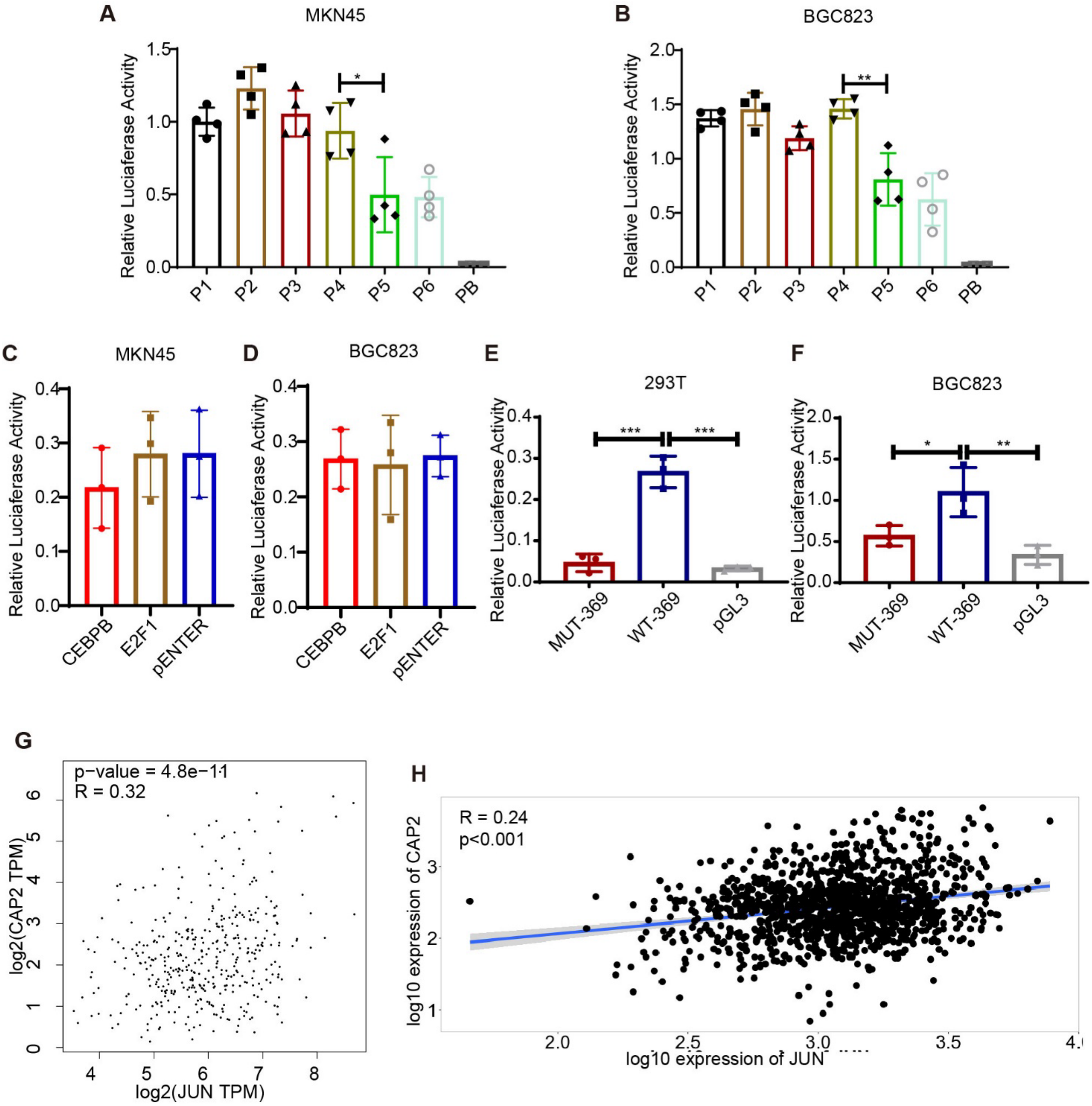

Supplemental Figure 2. JUN activates *CAP2* transcription. (**A and B**) Transcriptional activity of the putative *CAP2* promoter fragments in GC cells was analyzed by dual-luciferase reporter assays. (**C and D**) Dual-luciferase reporter assays showed that CEBPβ and E2F1 did not affect the promoter activity of pGL3-500/0 (n = 3). (**E and F**) Dual luciferase activity assays indicated that JUN binding mutants were unable to enhance pGL-500 promoter activity in HEK293-T and BGC823 cells (n = 3). (**G and H**) GEPIA (G) and TNMplot (H) analyses showed a positive correlation between *JUN* and *CAP2* expression. Data are presented as the mean ± SD. one-way ANOVA with Tukey's multiple comparison test (A-F). *$P < 0.05$, **$P < 0.01$, ***$P < 0.001$.

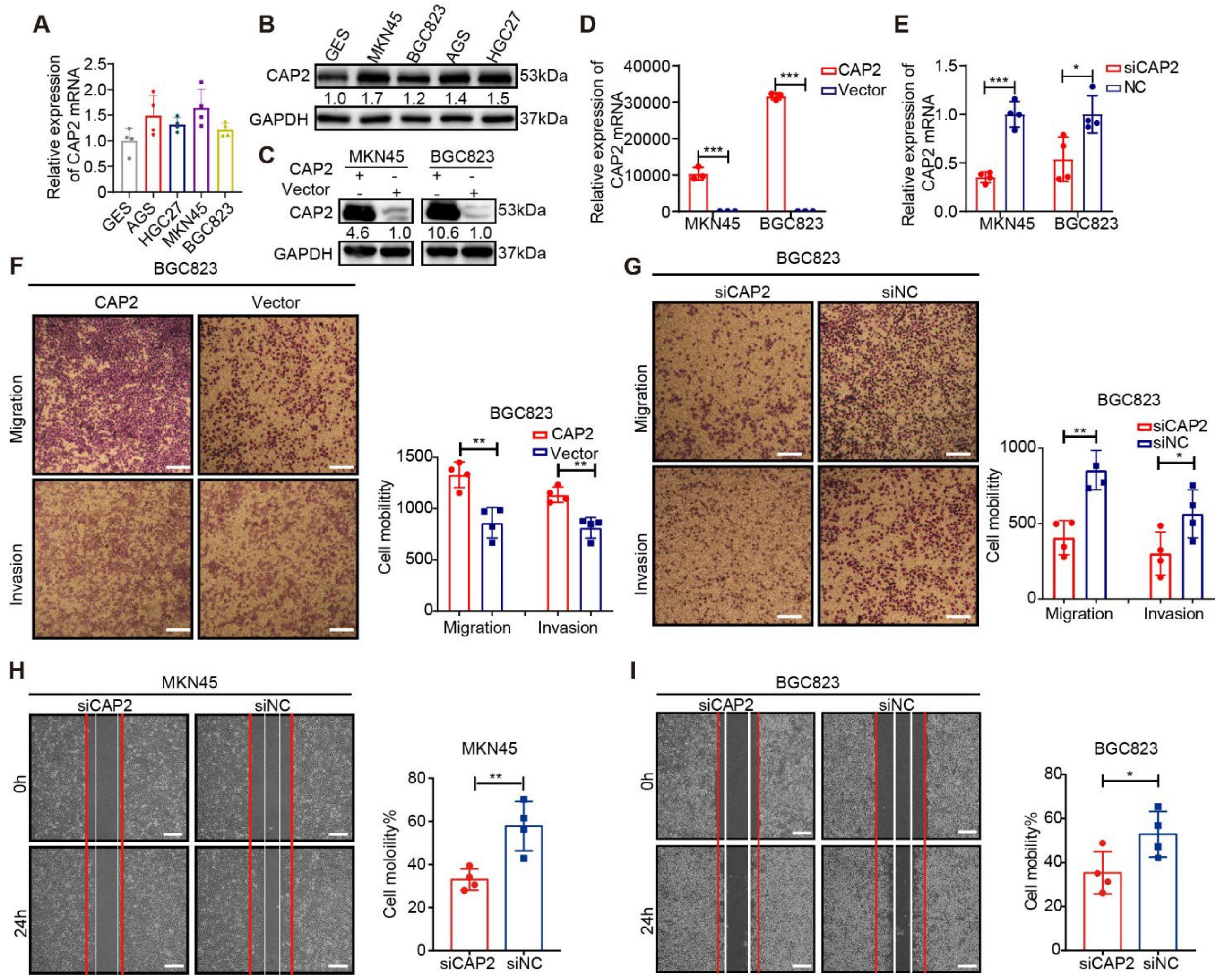

Supplemental Figure 3. CAP2 promotes GC progression. (**A**) qPCR detected mRNA expression of CAP2 in one immortalized gastric epithelial cell and four GC lines (*n* = 4). (**B**) The western blot experiment examined the protein expression of CAP2 in one immortalized gastric epithelial cell and four gastric cancer lines. (**C**) The overexpression efficiency of CAP2 was detected by western blot. (**D**) The overexpression efficiency of *CAP2* in GC cells was detected by RT-PCR (*n* = 4). (**E**) The knockdown efficiency of *CAP2* in GC cells was detected by RT-PCR (*n* = 4). (**F and G**) The migration and invasion ability of MKN45 cells was determined by Transwell assay. Scale bar: 200 μm, magnification ×40 (*n* = 4). (**H and I**) The migration ability of MKN45 and BGC823 cells was determined by a cell scratch test. Scale bar: 100 μm. (*n* = 4). Two-tailed unpaired Student's t test (D-I). *P < 0.05, **P < 0.01, ***P < 0.001.

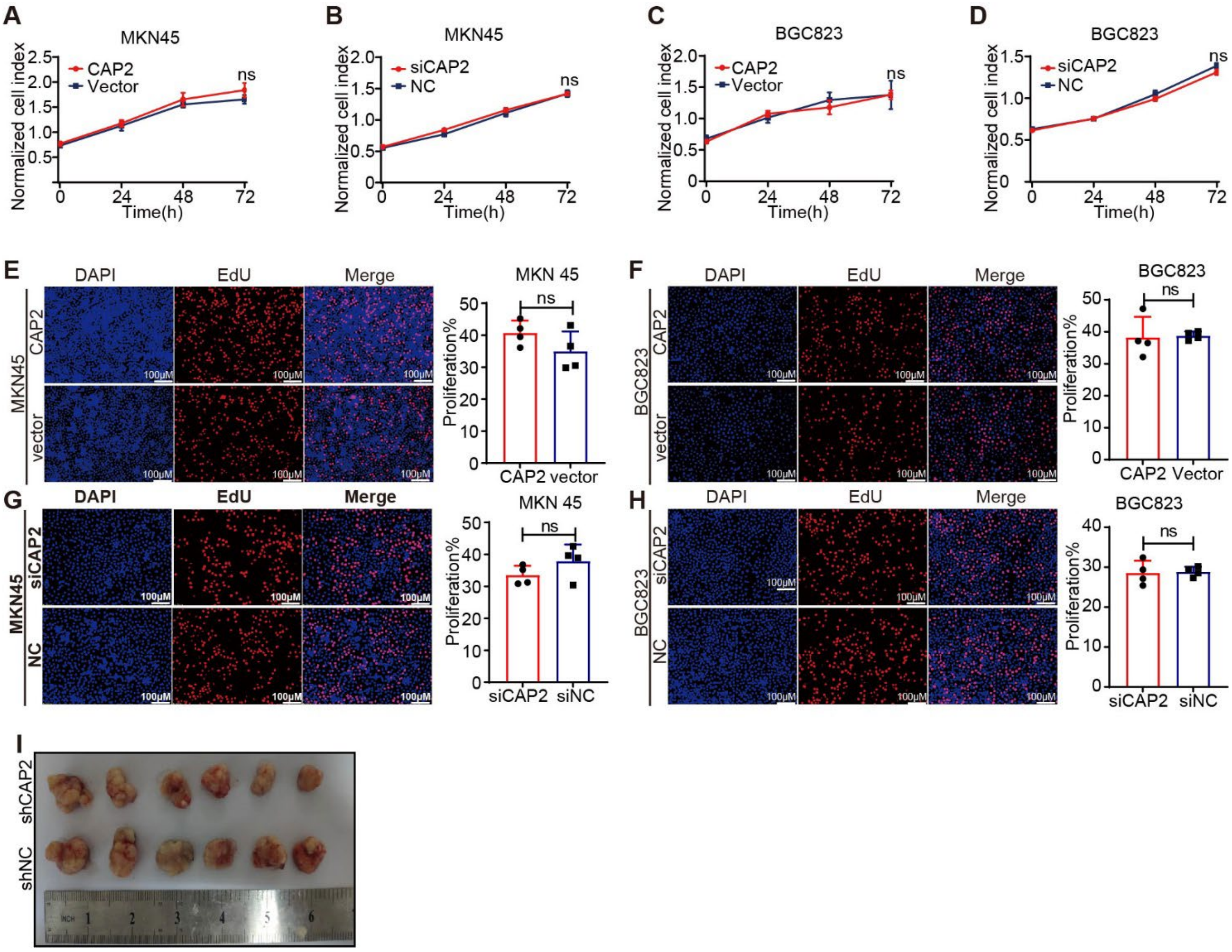

Supplemental Figure 4. The effect of CAP2 on gastric cancer proliferation. (**A-D**) Cell viability was measured using CCK8 assay in MKN45 and BGC823 cells. (**E-H**) Cell proliferation ability was detected by EdU(n = 3). Scale bar: 100 µm. (**I**) LV-shCAP2 or negative control was used for mouse subcutaneous tumorigenesis experiments. At 36 days after the subcutaneous injection, dissect the tumor and take pictures. Data are presented as the mean ± SD. ns, not significant. Two-tailed unpaired Student's t test (A-H).

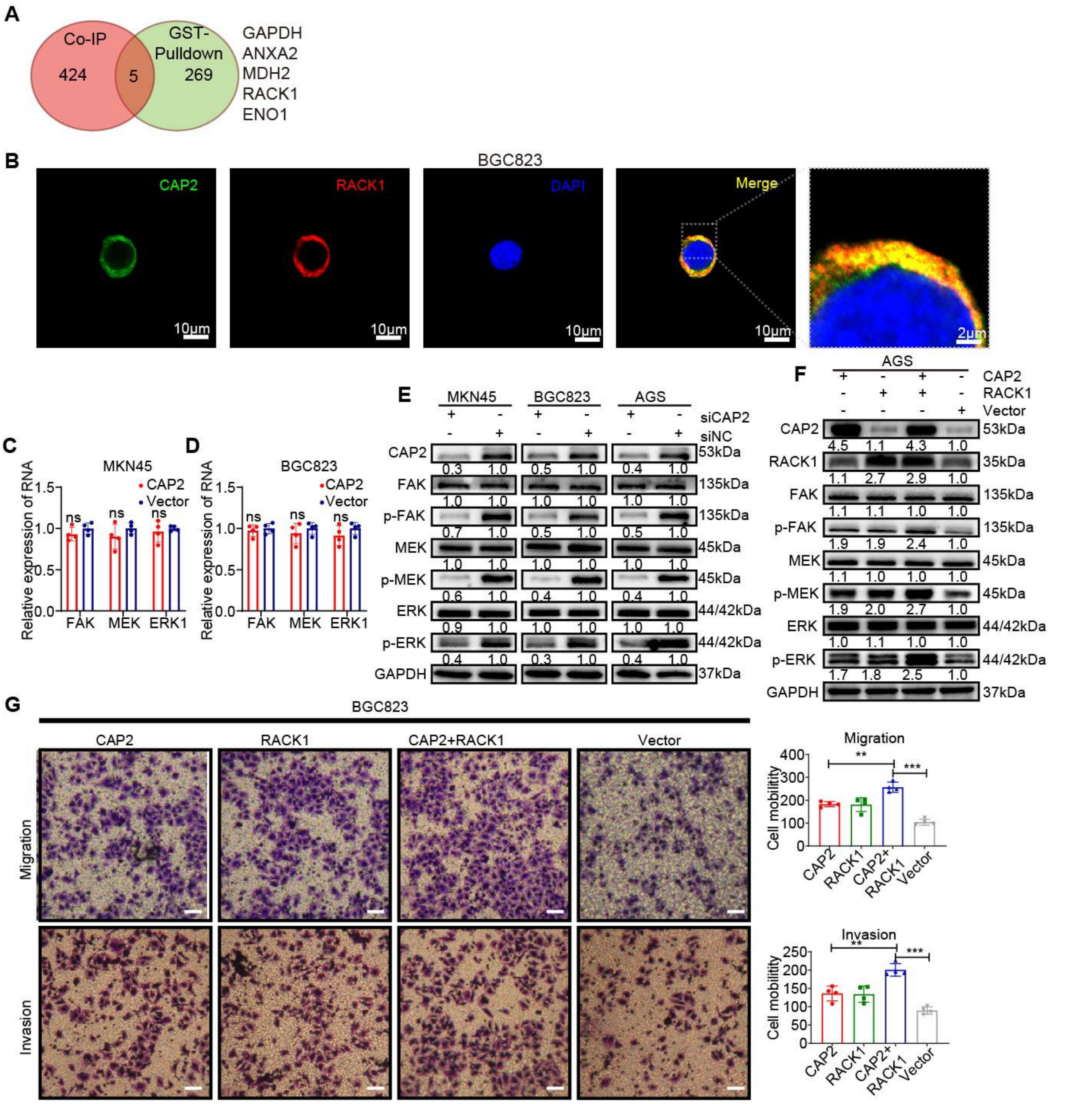

Supplemental Figure 5. CAP2 binds to RACK1 and activates the FAK/MEK/ERK axis. (**A**) Proteins obtained from co-IP ($n = 424$) and GST pulldown ($n = 269$). **(B)** Immunofluorescence analysis showed the CAP2/RACK1 colocalization in BGC823 cells. Scale bar: 10 μm; 2 μm(right). (**C** and **D)** The effect of CAP2 on the mRNA expression of *FAK*/*MEK*/*ERK* was detected by RT-PCR ($n = 4$). **(E)** Western blotting analysis of the FAK/MEK/ERK signaling after knockdown of CAP2 in GC cells. (**F**) Effects of CAP2 and RACK1 on FAK/MEK/ERK signaling were detected by Western blot. (**G**)**,** The migration ability of GC cell lines was determined by Transwell assay. Scale bar: 50 μm. Data are presented as the mean ± SD. Two-tailed unpaired Student's t test (C, D), one-way ANOVA with Tukey's multiple comparison test (G). ns, not significant. *$P < 0.05$, **$P < 0.01$, ***$P < 0.001$.

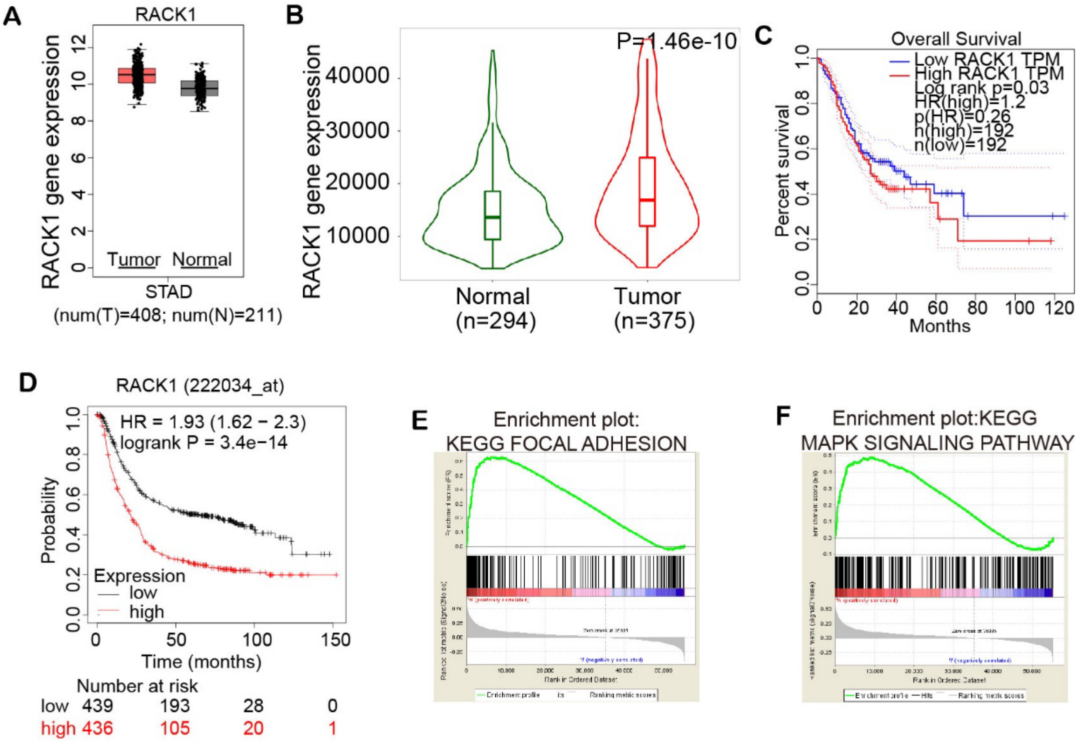

Supplemental Figure 6. Effects of RACK1 on gastric cancer. (**A** and **B**) GEPIA (E) and TNMplot (F) databases were used to analyze the expression of *RACK1* in gastric cancer and normal tissues. (**C**) GEPIA showed that patients with high expression of RACK1 had shorter overall survival. (**D)** The Kaplan–Meier Plotter showed that patients with high expression of RACK1 had poorer overall survival (http://kmplot.com/analysis/, P = 3.4e-14). (**E and F)** GSEA analysis showed that CAP2 expression was positively related with focal adhesion and MAPK signaling pathways.

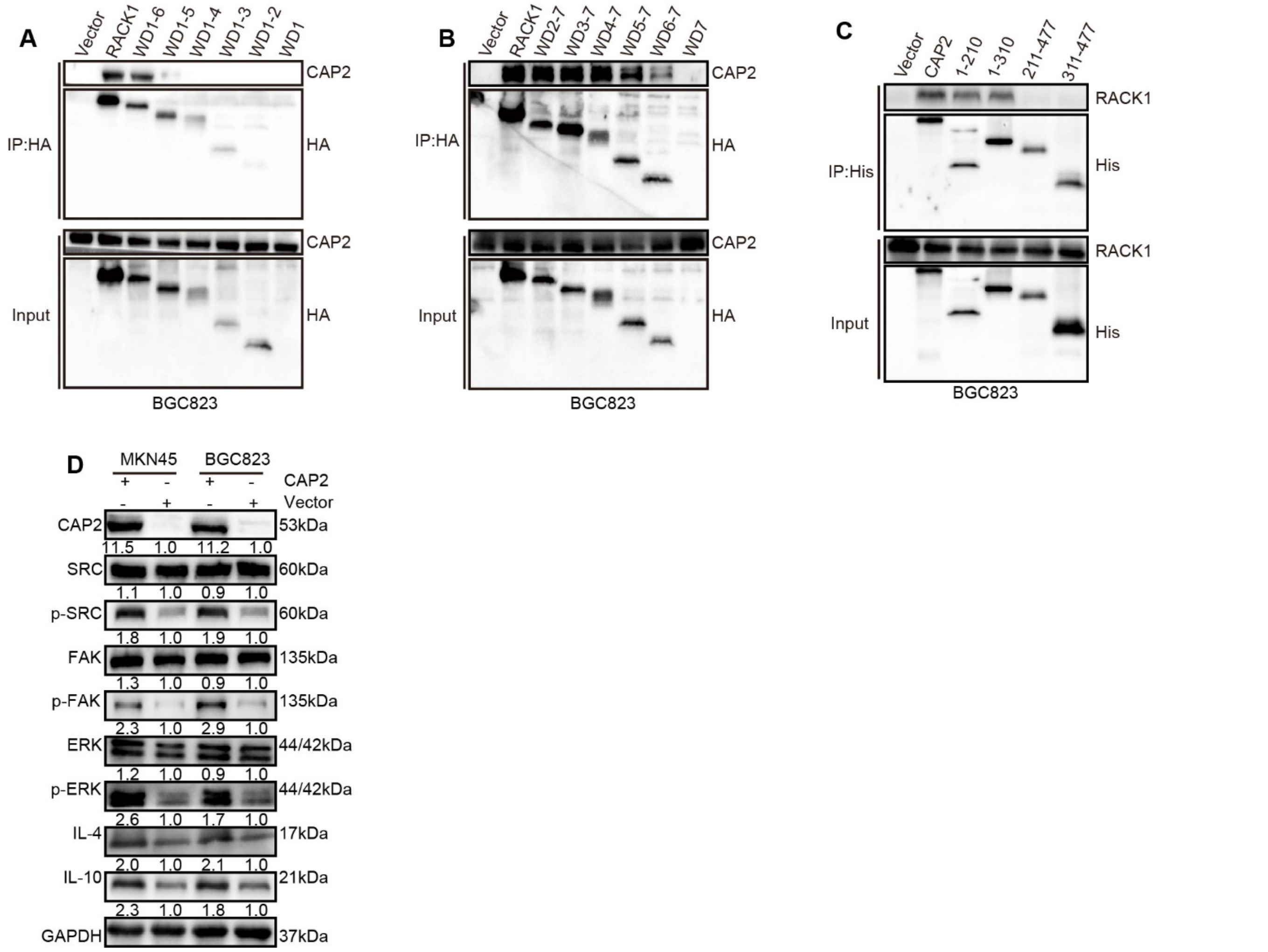

Supplemental Figure 7. CAP2 competitively binds to domains WD5 to WD7 of RACK1. (**A**) HA-tagged wt-RACK1, WD6-1, WD5-1, WD4-1, WD3-1, WD2-1, and WD1 were overexpressed in BGC823 cells, and anti-HA-tagged antibodies were used for co-immunoprecipitation. (**B**) HA-tagged wt-RACK1, WD2-7, WD3-7, WD4-7, WD5-7, WD6-7, and WD7 were overexpressed in BGC823 cells, and anti-HA-tagged antibodies were used for immunoprecipitation. (**C**) Overexpression of His-tagged wt-CAP2, CAP2(1-210bp), CAP2(1-310bp), CAP2(211-477bp), and CAP2(311-477bp) in BGC823 cells, immunoprecipitated with an anti-His-tag antibody. (**D**) Western blotting detected the expression and phosphorylation level of the SRC/FAK/ERK signaling pathway in GC cells with overexpression of CAP2.

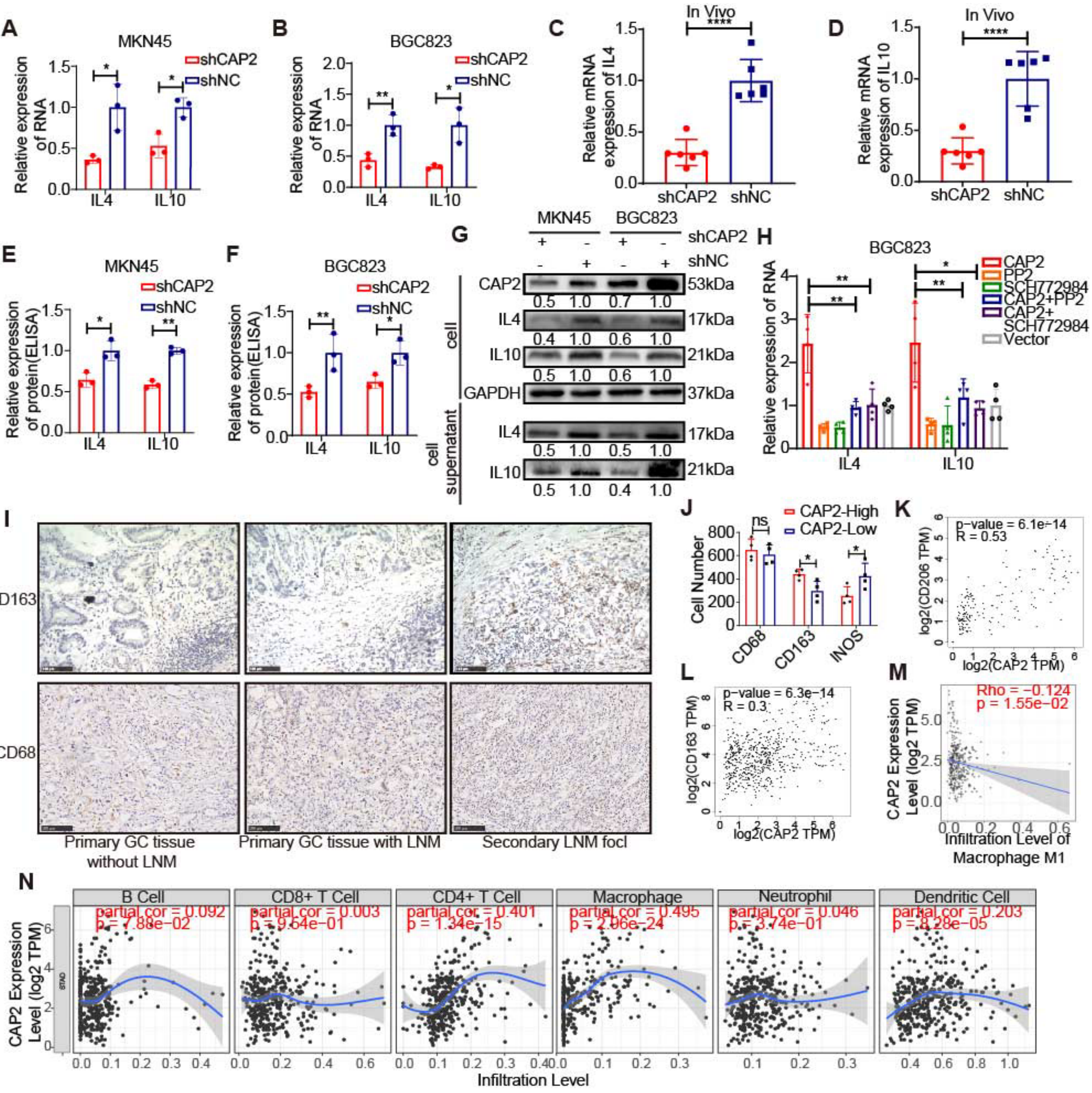

Supplemental Figure 8. CAP2 promotes IL4 and IL10 secretion by activating ERK. (**A and B**) The mRNA level of *IL4* and *IL10* in CAP2 knockdown was assessed using qPCR (*n* = 3). (**C and D**) The mRNA levels of *IL4* and *IL10* in xenografted tumors were assessed using PCR (*n* = 6). (**E and F**) Expression levels of IL4 and IL10 in the supernatant of GC cells were detected by ELISA (*n* = 3). (**G**) After the knockdown of CAP2, GC cell supernatants were concentrated using ultrafiltration tubes and subjected to Western blotting. (**H**) SRC inhibitor (PP2) and ERK inhibitor (SCH772984) were added to BGC823 cells, and the RNA levels of *IL4* and *IL10* were detected by qPCR (*n* = 4). (**I**) The expression of CD68 and CD163 in tissues was measured using IHC. Scale bar: 100 μm. (**J**) Number of CD68+, CD163+, and iNOS+ cells per field in tissues from GC patients

(n = 4). (**K**) The correlation between *CAP2* and *CD206* expression was analyzed with the GEPIA database. (**L**) The correlation between CAP2 and CD163 expression was analyzed with the GEPIA database. (**M**) Relationship between CAP2 and macrophage cell infiltration was analyzed using the TRIME2.0 website. (**N**) The correlation between CAP2 expression and immune cell infiltration was analyzed with TIMER 2.0. Data are presented as the mean ± SD. Two-tailed unpaired Student's t test (A-F, and J), one-way ANOVA with Tukey's multiple comparison test (H). *$P < 0.05$, **$P < 0.01$, ***$P < 0.001$.

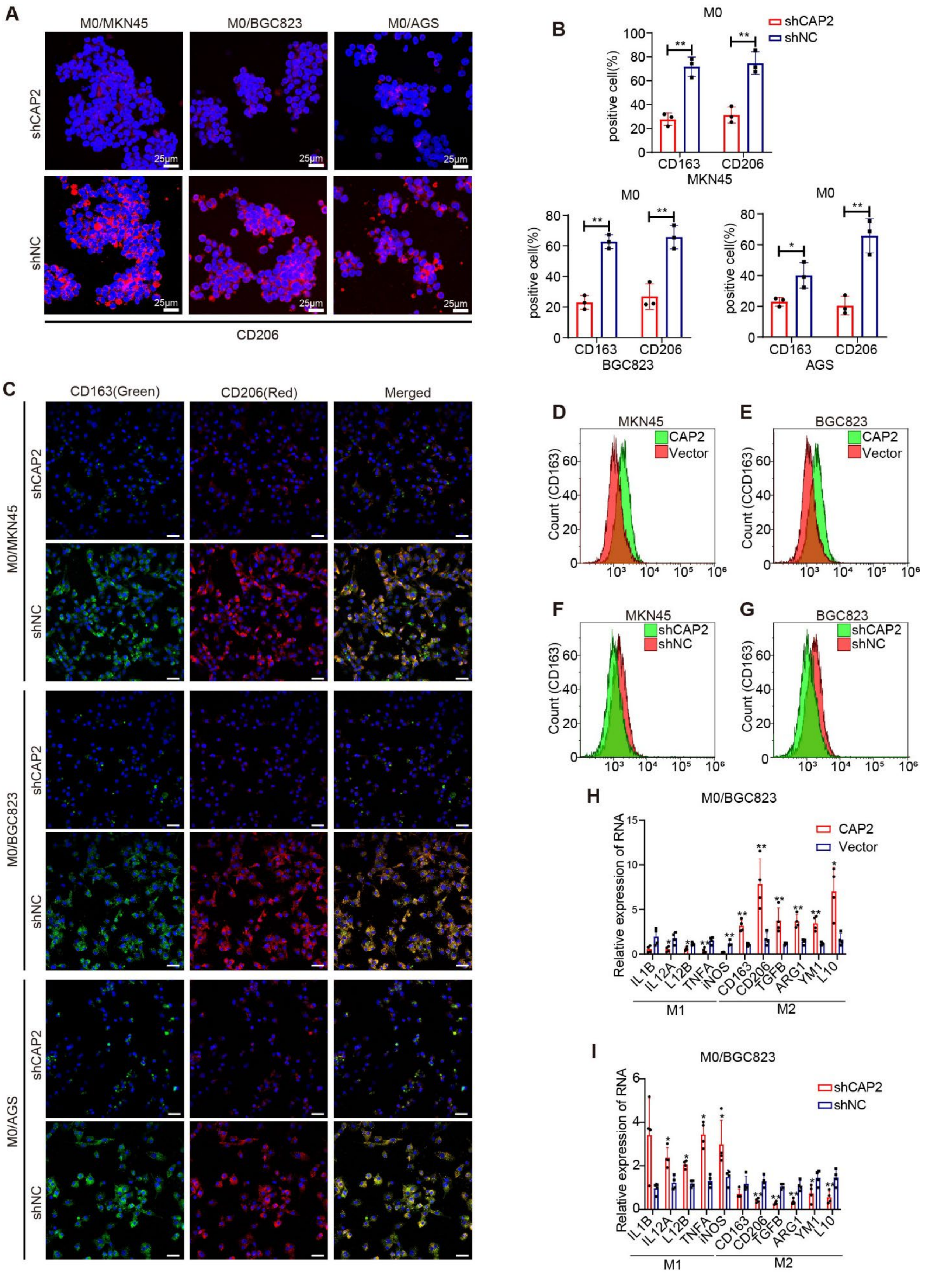

Supplemental Figure 9. CAP2 promotes macrophage polarization to the M2 phenotype. (A)

Expression of CD206 in macrophages was detected by immunofluorescence. Red staining indicates CD206 expression. Scale bar: 25 μm. (**B**) The quantitative data of CD163 and CD206 expression in immunofluorescence assays (*n* = 3). (**C**) The expression of CD163 and CD206 in macrophages was detected by immunofluorescence. Green staining indicates CD163; red staining indicates CD206; blue staining indicates DAPI (Scale bar: 50 μm). (**D–G**) Expression of CD163 in macrophages was detected using flow cytometry. (**H and I**) Expression of markers for M1 and M2 macrophages after co-culture of BGC823 cells and macrophages (*n* = 4). Data are presented as the mean ± SD. Two-tailed unpaired Student's t test (B, H, and I). $*P < 0.05$, $**P < 0.01$, $***P < 0.001$.

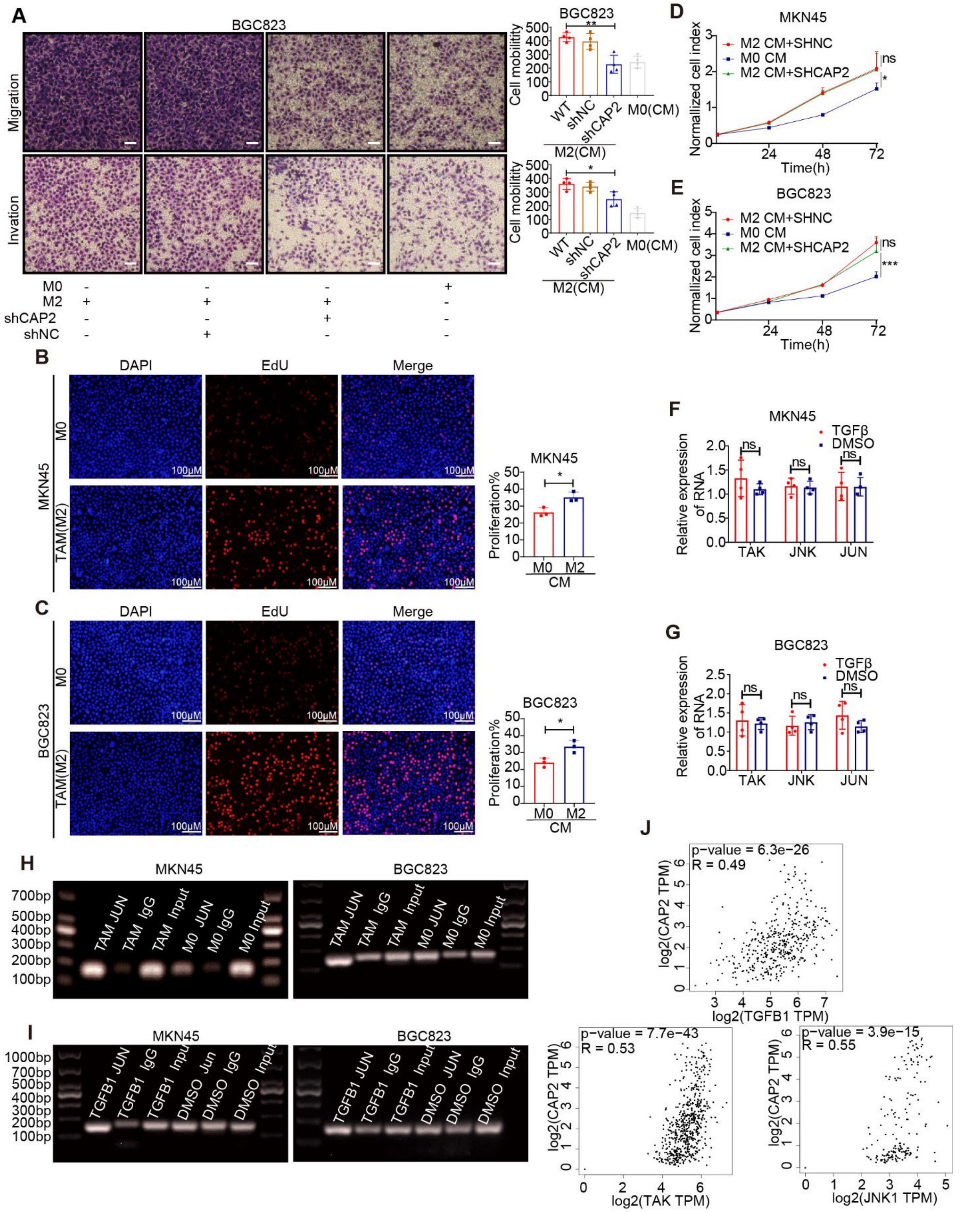

Supplemental Figure 10. TAMs promote CAP2 expression through TGFB1-mediated activation of JUN. (A) The migration and invasion abilities of GC cells were determined by Transwell assay after the GC cells were induced by TAM-conditioned medium (*n* = 4). Scale bar:

50 μm, (**B and C**) The cell proliferation abilities were detected by EdU after GC cells were induced by TAMs conditioned medium (Scale bar = 100 μm, $n = 3$). (**D and E**) The cell viability was detected by CCK-8 after GC cells were induced by TAM-conditioned medium ($n = 5$). (**F and G**) The mRNA expression of *TAK*/*JNK*/*JUN* were detected by RT-PCR after GC cells were treated with TGFB1 ($n = 4$). (**H and I**) DNA electropherogram for the ChIP assays. The binding ability of JUN to the *CAP2* promoter region was detected by ChIP after GC cells were treated with TGFB1 or induced by TAMs conditioned medium. (**J**) The correlation between *CAP2* and *TGFB1*, *TAK*, and *JNK* was analyzed by GEPIA. Data are presented as the mean ± SD. one-way ANOVA with Tukey's multiple comparison test (A, D, and E), Two-tailed unpaired Student's t test (B, C, F, and G). $^{*}P < 0.05$, $^{**}P < 0.01$, $^{***}P < 0.001$.

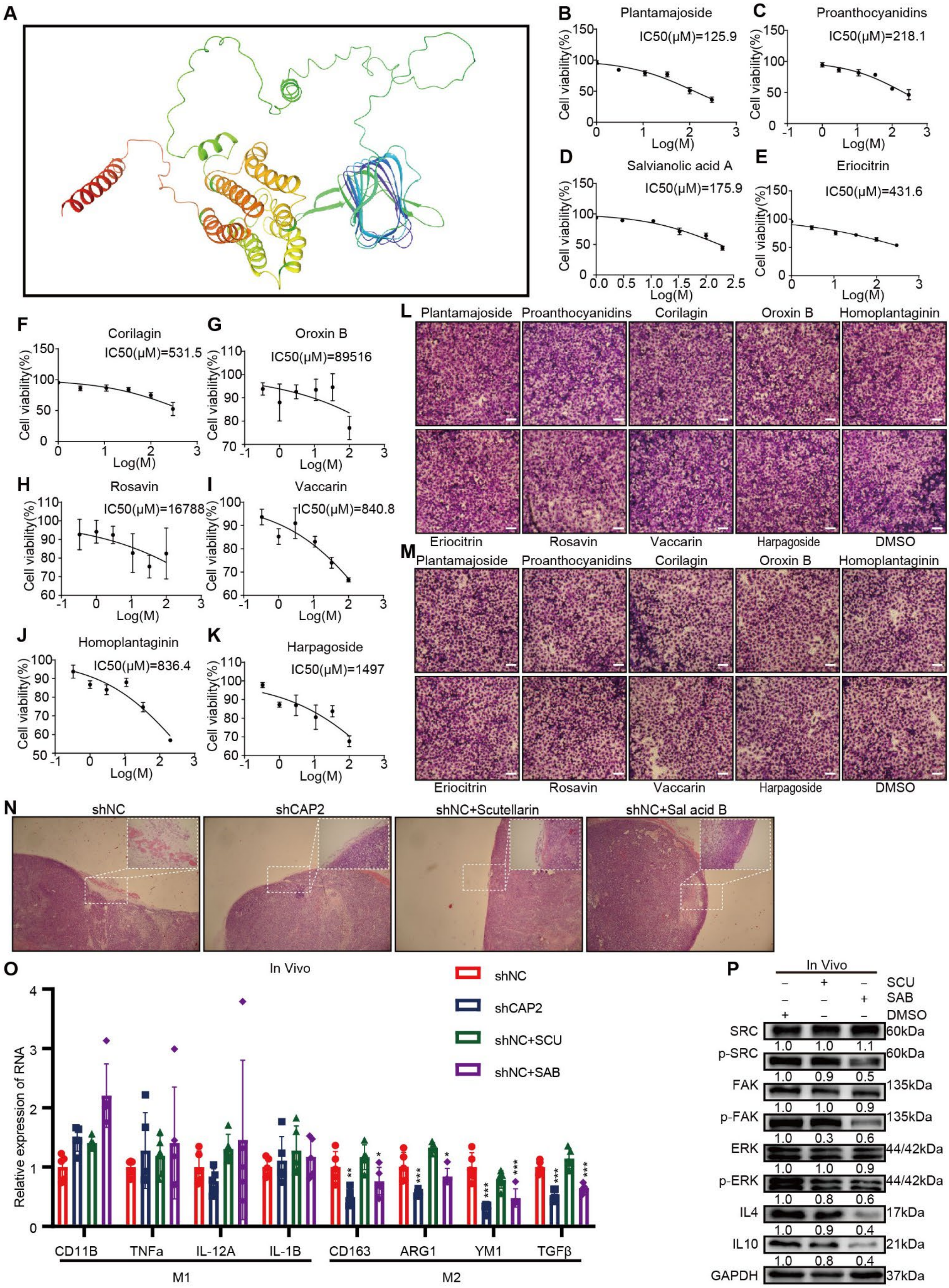
A
B
Plantamajoside
IC50(μM)=125.9
C
Proanthocyanidins
IC50(μM)=218.1
D
Salvianolic acid A
IC50(μM)=175.9
E
Eriocitrin
IC50(μM)=431.6
F
Corilagin
IC50(μM)=531.5
G
Oroxin B
IC50(μM)=89516
H
Rosavin
IC50(μM)=16788
I
Vaccarin
IC50(μM)=840.8
J
Homoplantaginin
IC50(μM)=836.4
K
Harpagoside
IC50(μM)=1497
Cell viability(%)
Log(M)
L
M
Plantamajoside
Proanthocyanidins
Corilagin
Oroxin B
Homoplantaginin
Eriocitrin
Rosavin
Vaccarin
Harpagoside
DMSO
N
shNC
shCAP2
shNC+Scutellarin
shNC+Sal acid B
O
In Vivo
shNC
shCAP2
shNC+SCU
shNC+SAB
Relative expression of RNA
CD11B
TNFa
IL-12A
IL-1B
CD163
ARG1
YM1
TGFβ
M1
M2
P
In Vivo
SCU
SAB
DMSO
SRC
p-SRC
FAK
p-FAK
ERK
p-ERK
IL4
IL10
GAPDH
60kDa
135kDa
44/42kDa
17kDa
21kDa
37kDa

Supplemental Figure 11. Screening and in vivo experiments of small molecule inhibitors. (**A**) The protein structure of CAP2 was predicted using AlphaFold. (**B-K**) Determination of IC50 of inhibitors on MKN45 cells. After treatment of gastric cancer cells with a series of doses (1, 3.5, 11, 33, 100, 300 μM) of inhibitors for 48 h, cell viability was determined by CCK8. (**L and M**) Transwell migration assay of MKN45 and BGC823 treated with inhibitor. Scale bar: 50 μm. (**N**) Mice subcutaneously injected with LV-NC MKN45 cells were treated with Scutellarin (SCU) and Salvianolic acid B (SAB), and then xenograft tumors were harvested. HE staining was used to observe tumor cell infiltration. (**O**) The expression of markers for infiltrating macrophages in the xenograft tumors was detected with qPCR ($n = 5$). (**N**) The expression of SRC/FAK/ERK/IL4/IL10 in the xenograft tumors was detected with western blot. Data are presented as the mean ± SD. one-way ANOVA with Tukey's multiple comparison test (O). *$P < 0.05$, **$P < 0.01$, ***$P < 0.001$.

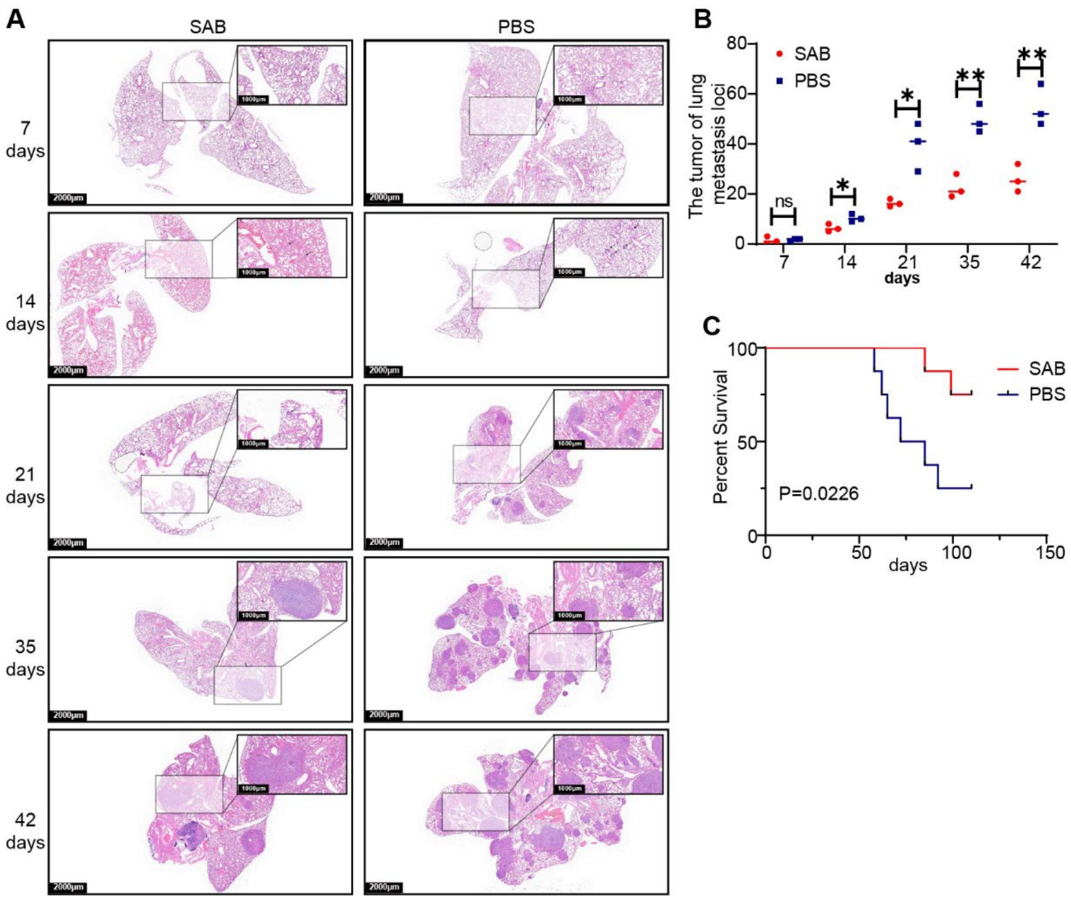

Supplemental Figure 12. Salvianolic acid B inhibits lung metastasis of GC cells. (**A**) Lung tissue was isolated 7, 14, 21, 35, and 42 days after tumor implantation for H&E staining to assess tumor metastasis. Scale bar: 2 mm; 1 mm (insets). (**B**) The number of transplanted tumors in the lungs of nude mice ($n = 3$). (**C**) The Kaplan–Meier analysis of animal endpoint survival. Each color represents a different experimental group: the SAB group (red, $n = 8$) and the control PBS group (blue, $n = 8$). *P < 0.05, Two-tailed unpaired Student's t test (B), logrank test (C). **$P < 0.01$, ***$P < 0.001$.

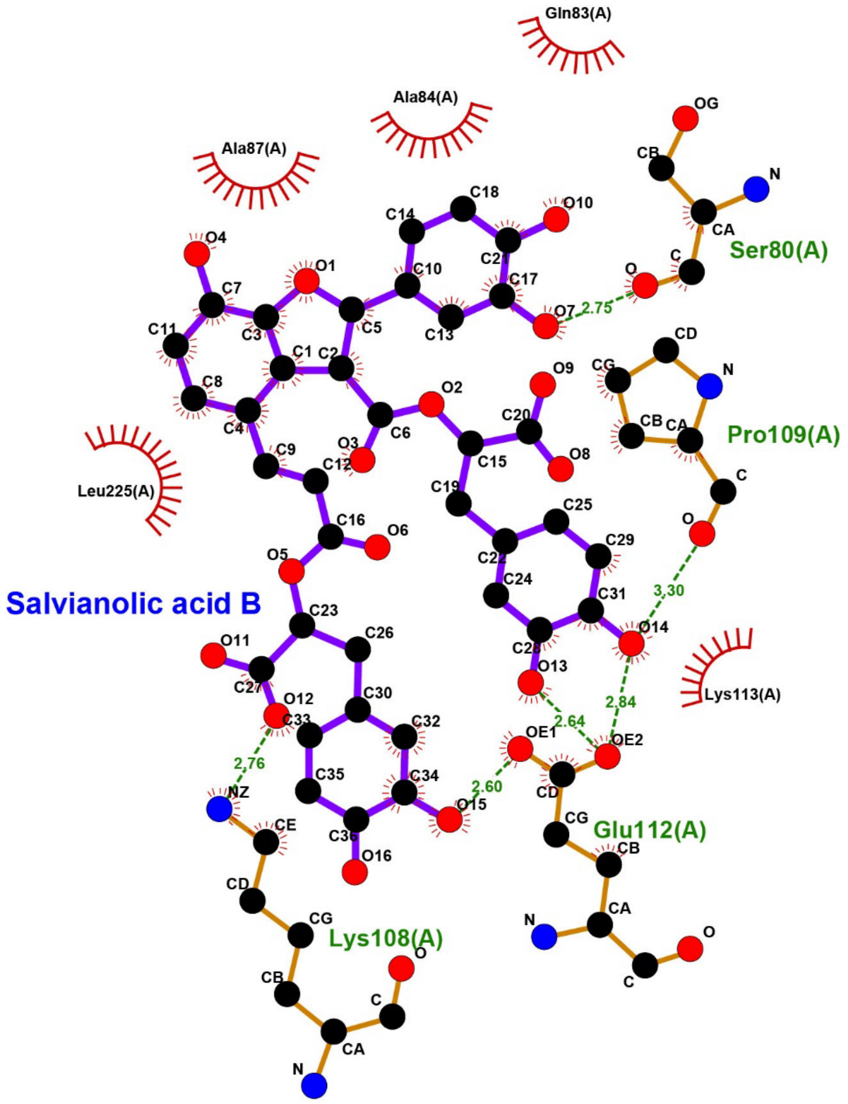

Salvianolic acid B+CAP2

Supplemental Figure 13. The schematic illustration for the binding between CAP2 and Salvianolic acid B.

Supplementary Materials and Methods

Cell proliferation assay

Cells were plated at 5000 cells/well in 96-well plates at 24, 48, and 72 hours after infection or conditioned medium (CM) induction using Cell Counting Kit-8 (CCK-8) (Solarbio, Beijing, China) Measure cell proliferation assay. Add 10 μL of CCK8 reagent to each well, incubate at 37°C for 2 hours, then measure the optical density at 450nM.

EdU experiment

2×10^4 GC cells transfected or co-cultured with macrophages were seeded into 96-well plates. After 12 hours, the EdU solution was added to the medium. Experiments were performed according to the instructions of EdU kit (Ribobio, Guangzhou, China). Cells were incubated with EdU reaction mixture and counterstained with DAPI. Observe and record using a fluorescence microscope.

Induction of macrophages and co-culture of cells

To obtain mature macrophages (M0), THP1 cells were added with 100 ng/ml of phorbol 12-myristate 13-acetate (MCE, New Jersey, USA) and induced for 48 hours. M2 macrophages were obtained by incubating M0 macrophages in a complete medium containing 20 ng/ml of IL4 and 20 ng/ml of IL13 for 48 hours. The co-culture system of GC and macrophages used the Transwell system (Corning, Corning, USA). 3×10^5 GC cells were added to the upper chamber, and an equal amount of M0 or M2 macrophages were added to the lower chamber. After 48 hours, cells in the upper and lower chambers were collected for subsequent experiments.

Co-immunoprecipitation

Experiments were performed according to the instructions using a co-immunoprecipitation kit (Cat. 26149, Thermo Fisher). First use anti-CAP2 (2 μg, Ct:15865; Proteintech), anti-RACK1 (2 μg, Ct:27592; Proteintech), anti-HA (2 μg, Ct:3724; CST) or anti-His (2 μg , Ct:12698; CST) for antibody immobilization followed by incubation with GC cell lysates at 4°C overnight. Bound protein was eluted and detected by Western blot.

Immunofluorescence

Fixed with 4% PFA and permeabilized with 0.1% Triton X-100. The primary antibody used is shown in Supplemental Table 4. Counterstaining with A488-labeled (1:100, SA00013-2, Proteintech) and A594-labeled (1:100, SA00013-3, Proteintech) secondary antibodies. Incubate overnight with the primary antibody at +4°C and with the secondary antibody for 30 min at room temperature. Immunofluorescence images were obtained by fluorescence confocal microscopy (Zeiss LSM 880).

Small molecule compound cytotoxicity assay

Selected small molecule compounds were doubling dilutions (1, 3.5, 11, 33, 100, and 300 μM) in a complete medium, and then MKN45 cells were cultured. After 48 hours, cell viability was determined by CCK8 assay. IC50 values were calculated using GraphPad Prism 9.

In *vivo* experiments with small molecule inhibitors

For tumor-killing experiments, 4×10^5 MKN45 cells were inoculated into the left axilla of 4-week-old male BALB/c nude mice (Vital River, Beijing, China). After GC cell seeding, local injections of drugs were administered for 36 days: SAB treatment (80 mg·kg−1·day−1), SCU treatment (40

mg·kg−1·day−1), and no treatment (PBS/day). Tumor volume is measured once a week. Mice were injected with 4 × 105 MKN45 cells via their tail veins. All of the animals were then divided into two groups (23 in each group) and injected with either SAB (80 mg·kg−1·day−1) or PBS via their tail veins the day after GC cell seeding. Mice were euthanized on days 7, 14, 21, 35, and 42 (three per group), and H&E staining of lung sections was performed to check the lung metastasis status of mice. The monitoring of the survival status of the remaining mice was continued until the endpoint.
